# Recent Progress in Anion Exchange Membrane Water Electrolysis: From Membrane Materials to System Components

**DOI:** 10.3390/membranes16060185

**Published:** 2026-05-28

**Authors:** Adil Emin, Jiarui Liu, Xian Sun

**Affiliations:** School of Chemical Engineering, Xinjiang University, Urumqi 830017, China; 18191859848@163.com

**Keywords:** hydrogen production by green electricity, anion exchange membrane water electrolysis, ionic conduction mechanism, ion conductivity

## Abstract

Hydrogen energy, as an important green energy source, is a crucial guarantee for achieving carbon neutrality and peak carbon emission. The anion exchange membrane (AEM) electrolysis cell combines the advantages of alkaline electrolysis cell and proton exchange membrane electrolysis cell and can employ non-precious metal catalysts combined with renewable energy, which is expected to break through the bottleneck of high production cost of green hydrogen. AEM water electrolysis combines the advantages of alkaline and proton exchange membrane water electrolysis for hydrogen production. It has the characteristics of high electrolysis efficiency, fast response rates, and low cost, and its considered one of the most promising renewable green energy hydrogen production technologies at present. AEM is a key component that provides OH^–^ ion conduction and blocks gas crossover, which directly affects the performance and service life of the AEM electrolysis water system. However, current AEMs face issues of low ion conductivity and poor stability. This review introduces the role of AEM in electrolytic cells, the performance requirements and evaluation parameters that high-performance AEM should meet, and focuses on the transport mechanism and influencing factors of OH^–^ in AEM. Furthermore, this review provides an overview of the structural composition of AEM, as well as common cationic groups and polymer backbone types. The degradation mechanism of various cationic groups and the characteristics of polymer main chains were elaborated, with a focus on the strategies for designing the stability of cationic functional groups, the methods for modifying and preparing polymer main chains, and the performance of AEMs. Finally, the future challenges and potential research directions of AEM membranes are discussed. It is suggested that high-performance AEMs meeting practical application needs should be developed through strategies such as crosslinking, block copolymerization, side chain grafting, and composite membrane technology, based on the design of alkali-resistant and stable AEM membranes. These insights provide reference and guidance for the further development of AEMs.

## 1. Introduction

With the rapid depletion of global fossil fuels, the greenhouse effect and environmental pollution are becoming increasingly severe, and reducing dependence on carbon-based fossil fuels is facing unprecedented urgency. However, due to the intermittent and regional nature of green renewable energy sources such as wind, tidal and solar energy, large-scale energy storage and transmission capabilities are required. Hydrogen has the advantages of high energy density, zero carbon emissions, and non-toxicity, making it an ideal energy carrier for building a global renewable energy system. However, as a secondary energy source, over 95% of H_2_ in the world is produced through high-temperature reforming of natural gas [1,2,3,4,5]. This is a non-renewable high carbon emission process that produces at least 5.5 kg CO_2_ for every 1.0 kg of H_2_ produced [3,4,5,6]. Electrolyzing water to produce hydrogen not only solves the intermittent problem of solar and wind energy, but also improves the utilization rate of renewable energy, making it one of the most promising solutions currently available.

According to the type of membrane used, the current hydrogen production technology through electrolysis of water can be divided into alkaline water electrolysis (AWE) technology, proton exchange membrane water electrolysis (PEMWE) technology, and anion exchange membrane aqueous electrolysis (AEMWE) technology (Table 1) [7,8,9]. AWE technology uses nickel-based alloys as electrodes, KOH solution as electrolytes, and polyphenylene sulfide woven fabric as separators, which has the advantages of low cost and high technological maturity. However, the energy efficiency of this technology is low (70% to 80%), the purity of hydrogen production is low (>99.5%, 3 MPa), the current density is low, and the dynamic response capability is poor, resulting in poor compatibility with renewable energy [10,11,12,13,14,15]. PEMWE technology uses perfluorosulfonic acid proton exchange membranes (such as Nafion membranes) as separators, reducing ion conductivity resistance and improving electrolysis efficiency, and can operate at high current densities (>2 A/cm^2^). At the same time, PEMWE also has fast dynamic response capability and high compatibility with renewable energy [16,17,18,19,20,21]. However, PEMWE requires the use of precious metal electrocatalysts, such as platinum-based electrocatalysts for hydrogen evolution reactions at the cathode and iridium- or ruthenium-based electrocatalysts for oxygen evolution reactions at the anode [22,23,24,25,26,27]. Moreover, the higher the working current density, the higher the required loading of precious metal catalysts [26,27,28]. Due to the influence of device costs such as precious metal catalysts, Nafion membranes, and porous transport layers, the cost of PEMWE stacks is higher than that of AWE stacks, which seriously hinders the large-scale application of PEMWE. AEMWE technology combines the advantages of AWE and PEMWE while overcoming their limitations [27,28,29,30,31,32,33]. AEMWE uses anion exchange membrane (AEM) as the membrane, which can achieve high working current density and high electrolysis efficiency (>90%), with the ability to respond quickly and minimize the mechanical compression requirements for storing hydrogen. Meanwhile, this technology can operate in weakly alkaline environments and can use low-cost and resource-rich metals such as nickel as electrocatalysts. The characteristics possessed by AEMWE have shown great application prospects in large-scale sustainable hydrogen production, and have also attracted the attention and investment of more scholars and entrepreneurs [34,35,36,37,38]. However, the main challenges currently faced by AEMWE technology are the poor stability and low ionic conductivity of AEM in alkaline environments [39]. Therefore, finding ways to develop high-performance AEMs is crucial for fully leveraging the application of AEMWE technology in large-scale, safe, efficient, and green ecological hydrogen production.

This review first briefly outlines the composition, working mechanism, and performance requirements of AEMs. Subsequently, the types of AEM cationic groups, degradation mechanisms, and modification design strategies were discussed. The types, structural design, and modification preparation methods of polymer main chain structures were also discussed. Finally, the challenges faced by current AEM research were summarized, and potential future research directions were proposed to provide reference for future researchers.

### 1.1. AEMWE Hydrogen Production Technology

#### 1.1.1. Principles of AEMWE Hydrogen Production Technology

**Figure 1:**
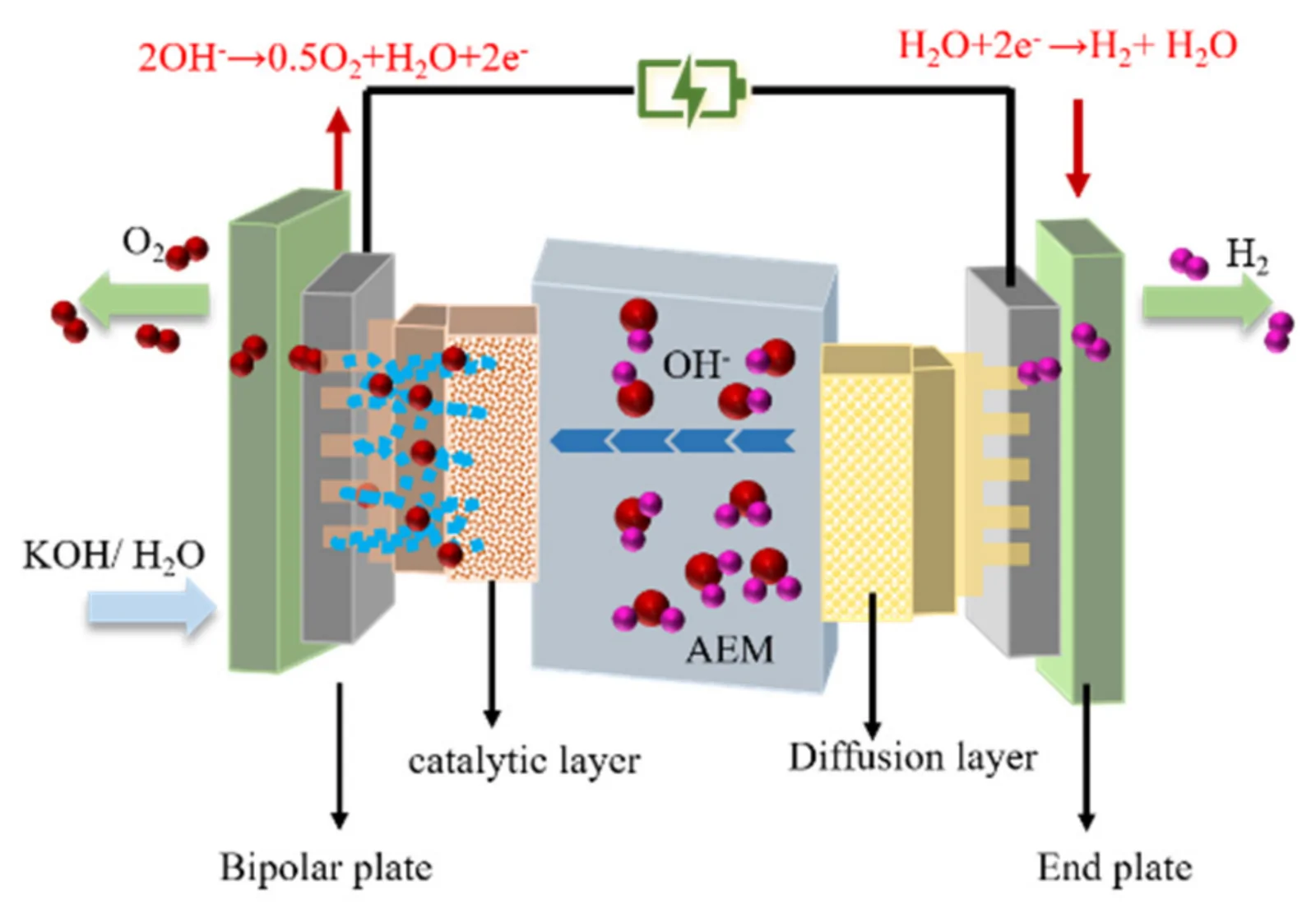
Simplified diagram of AEMWE [11].

AEMWE hydrogen production technology was developed based on AWE and PEMWE technologies. Its structure is similar to that of a PEM electrolyzer, but uses an AEM instead of a PEM for OH^−^ transport, as shown in Figure 1. AEMWE combines the advantages of AWE and PEMWE. Operating under weakly alkaline conditions, it allows for the use of inexpensive non-precious metal catalysts, thereby reducing catalyst costs and energy consumption. Furthermore, the costs of the AEM, porous transport layer, and bipolar plates are all lower than those of their counterparts in PEMWE, enabling significant cost reductions. At the same time, the use of a polymer membrane ensures excellent dynamic response characteristics, similar to PEMWE technology, making it well-suited for the fluctuations of renewable energy sources. Low cost and high dynamic response are the primary advantages of AEMWE. Furthermore, AEMWE can use pure aqueous or low-concentration alkaline solutions as electrolytes instead of concentrated KOH solutions, effectively avoiding severe corrosion issues. As a result, the entire water electrolysis system offers benefits such as leak-free operation, compact size, and ease of maintenance. With its low overall hydrogen production cost and high stability, it is well-suited for large-scale hydrogen production using renewable energy [38,39,40,41,42].

An AEM electrolyzer primarily consists of an AEM, a catalyst layer (CL), a gas diffusion layer (GDL), and a sealing layer such as BP [43,44,45,46]. The operating principle is similar to that of other water electrolysis methods for hydrogen production and involves two half-reactions: the oxygen evolution reaction (OER) and the hydrogen evolution reaction (HER). Using pure aqueous or a low-concentration alkaline solution as the electrolyte, the electrolyzer electrochemically decomposes water to produce oxygen and hydrogen under a voltage of typically 1.8 to 2.5 V. The key to the technology is the AEM, which transports OH^−^ from the cathode to the anode while preventing the direct transfer of gas and electrons between the electrodes. Due to the inert reaction kinetics of the OER and HER processes, it is typically necessary to load a catalyst onto the electrodes to enhance reaction activity and reduce energy consumption [47,48,49,50].

The alkaline water-splitting reaction and its thermodynamic potential are shown below [13].

$$\mathrm{Cathode}:\;4H_{2}O+4e^{-}\to 2H_{2}+4OH^{-}\;\;\;\;\;\;\;\;\;E^{o}=-0.828\;V$$

$$\mathrm{Anode}:\;4OH^{-}\to O_{2}+2H_{2}O+4e^{-}\;\;\;\;\;\;\;\;\;\;\;\;\;\;\;E^{o}=0.401\;V$$

$$\mathrm{Overall}\;\mathrm{reaction}:\;2H_{2}O\to 2H_{2}+O_{2}\;\;\;\;\;\;\;\;\;\;\;\;\;\;\;E^{o}=-0.427\;V$$

Under alkaline conditions, two water molecules are required for each H_2_ produced at the cathode; therefore, water transport from the anode to the cathode is a critical factor that must be considered in cell design, material selection, and operating modes. The OH^−^ required at the anode is supplied by the cathode reaction and must be transported through the catalyst layer and membrane to the active sites in the anode layer; thus, the conductivity of the AEM is crucial for electrolysis efficiency [13,51,52,53]. *E*_0_ is the reversible potential; under standard conditions (pressure *P* = 0.1 MPa, temperature *T* = 298.15 K), the reversible cell voltage for water splitting is 1.23 V. However, during the actual operation of an AEM electrolyzer, irreversible losses occur as current flows through the cell, and the actual voltage of the water-splitting reaction is often higher than the reversible cell voltage. The actual operating voltage of an AEM electrolyzer is primarily composed of four components: the open-circuit voltage *E*_ocv_, the activation overpotential *V*_Act_ at the anode and cathode, the diffusion overpotential *V*_diff_, and the ohmic overpotential *V*_ohm_, as shown in Equation (1) [13].

(1) $$V=E_{ocv}+V_{Act}+V_{diff}+V_{ohm}$$

The open-circuit voltage, also known as the minimum theoretical voltage, represents a potential loss in electrochemical reactions caused by the activation overpotential, which is influenced by physical and chemical parameters such as catalyst performance, cell temperature, and electrode morphology [54,55,56,57,58]. The diffusion overpotential arises from mass transport within the porous electrode; at high current densities, reaction-generated bubbles can block the active area, disrupt the contact between the electrode and the electrolyte, and reduce catalyst utilization. The ohmic overpotential is primarily caused by bipolar plate resistance, electrode resistance, membrane resistance, and interfacial resistance between different layers; for current AEMWEs, membrane resistance is the dominant factor [12]. Vincent et al. [59] found that maintaining an appropriate electrolyte flow rate helps reduce ohmic resistance.

#### 1.1.2. Analysis of the Current Status of AEMWE Hydrogen Production Technology

As an emerging water electrolysis technology, AEMWE is still in the early stages of research. According to an IEA report, the current technology levels for AWE and PEMWE are both at Level 9 (market uptake), while AEMWE technology has risen from Level 4 (small prototype) in 2021 to Level 6 (large prototype) in 2022 [14,15,16,17,18]. It is believed that with increased research and development denoted as R&D investment, AEMWE technology will continue to mature. However, the technology currently faces several unresolved technical bottlenecks, such as the AEM’s weak ionic conductivity, thermal and chemical stability that have not yet reached commercial levels, and insufficient activity of non-precious metal-based catalysts. Furthermore, during prolonged high-temperature reactions, the catalysts in the membrane electrode assembly are prone to dissolution and detachment, resulting in poor overall electrolysis performance of the device. To address these issues, current research is primarily focused on developing high-performance non-precious metal catalysts, enhancing membrane stability and ionic conductivity, and gaining a deeper understanding of the operational mechanisms of the AEMWE system.

Table 2 summarizes the International Renewable Energy Agency (IRENA)’s techno-economic targets for AEMWE. To become competitive and meet the 2050 targets, breakthroughs in performance, durability, and cost are urgently needed. Recent studies in this area have also yielded promising results: in terms of electrolysis performance, an AEM electrolyzer using a platinum group catalyst achieved a current density of 7.68 A/cm^2^ using a current density in a 1 mol/L KOH electrolyte at 2.0 V [12,13,14]. In terms of stability, an AEM electrolyzer using the ether-free Sustainion membrane operated stably for 12,000 h at 60 °C in a 1 mol/L KOH electrolyte, meeting industrial performance requirements [37]. These outstanding results foreshadow the potential for a technological breakthrough in AEMWE.

However, current research on AEMWE has focused primarily on the development of AEMs, anodes, and cathode catalysts, with only a few studies addressing system integration. The performance of individual membranes and catalysts in test systems does not necessarily translate to the same results when integrated and encapsulated within an electrolyzer. Of course, research on integrated electrolyzers inevitably requires more time, funding, and human resources, but this is an essential step toward the industrialization of AEMWE. Furthermore, to date, no reasonable standards have been established for performance evaluation, cost estimation, gas purity, or operational mechanisms [9,38,39,40,41,42,43]. In summary, the R&D of AEMWE technology faces a long and arduous journey. The current research priorities and challenges for AEMWE technology include gaining a comprehensive understanding of the characteristics and mechanisms of all components within the AEMWE system, establishing a standardized performance evaluation system, developing high-performance system components, and advancing research on integrated electrolyzers.

## 2. Research Progress on Key Components of AEM Electrolyzers

An AEM single cell typically consists of bipolar plates and a membrane electrode assembly (MEA). The MEA serves as the site of electrochemical reactions and is the key component determining the cell’s performance; it generally comprises an AEM, a polymer, an anode, a cathode catalyst layer, and a porous layer. In addition to the MEA, bipolar plates and electrolytes are also key areas of research in AEMWE technology. Therefore, research on the key components of AEM fuel cells is divided into the following eight aspects.

### 2.1. Anion Exchange Membranes

Anion exchange membranes are one of the core components of AEM electrolyzers, serving two primary functions: (1) acting as internal channels to conduct OH^−^ and (2) isolating hydrogen produced at the cathode from oxygen produced at the anode to prevent hazardous incidents. AEMs play a critical role in the overall performance and durability of the electrolyzer. The microstructure of an AEM typically consists of various cationic groups and a polymer backbone; these cationic groups confer anion selectivity to the membrane. Among these, quaternary ammonium groups are most commonly used [17], while the polymer backbone is typically composed of polyarylether, polystyrene, polysulfone, polyethersulfone, or polyoxymethylene, among others [18,19,20,60,61,62,63,64,65]. Existing research indicates that the polymer backbone structure primarily influences mechanical and thermal stability, while cationic groups mainly affect ion exchange capacity, ionic conductivity, and transport numbers. The polymer backbone and cationic groups jointly determine chemical stability; therefore, the overall polymer structure has a certain impact on both the degradation mechanism and degradation rate of the membrane [18,42,43,44,45,46]. As the most advanced aqueous electrolysis technology currently available, the AEMWE system has a relatively short development history, and research on high-performance AEMs remains in the early exploratory stage. Compared to the technologically mature PEM, AEMs exhibit lower ionic conductivity and chemical stability than perfluorosulfonic acid membranes. In terms of conductivity, compared to PEM, AEM exhibits lower OH^−^ conductivity, approximately half that of H^+^ [21]; regarding stability, under alkaline conditions and at temperatures of 60–80 °C, the polymer backbone and organic cationic groups are susceptible to OH^−^ attack, leading to chemical degradation and a sharp decline in the membrane’s mechanical properties and ionic conductivity [20,66,67,68,69,70]. Consequently, the relatively low ionic conductivity and limited durability of AEMs have long been major obstacles to the large-scale adoption of AEMWE [22,45,46,47,48,49,50]. To address these issues, researchers have conducted the following studies on membrane materials and structures.

#### 2.1.1. Strategies for Enhancing Ion Conductivity in AEMs

Microphase separation is considered a nanoscale phenomenon in polymer electrolyte membranes, characterized by the formation of hydrophobic and hydrophilic regions containing ionic groups [23,71,72,73]. The ionic hydrophilic regions can absorb and aggregate a large number of water molecules, while simultaneously interconnecting to form ion transport channels. Well-connected water channels effectively promote ion conduction, thereby enhancing ionic conductivity without causing excessive swelling. To date, years of research have yielded various strategies for inducing microphase separation in AEMs [24,60]. Lu et al. [74] successfully prepared cross-linked AEMs using polybenzimidazole as the polymer crosslinking agent and *N*-methylpiperidine-quaternized polyvinylbenzyl chloride as the ion-conducting component.

In the resulting PBI1-PVBC1-NMPD/OH membrane, continuous ion transport channels were formed via microphase separation, with a hydroxide conductivity exceeding 80 mS/cm at 80 °C. Wu et al. [39] formed hexagonal ion channels through the coordination of Cu^2+^ with chitosan chains via amino and hydroxyl groups, providing space for water diffusion and ion transport. This design approach can inspire the synthesis of many ion exchange membranes for ion transport, ion sieving, and ion filtration. Simari et al. [40] first proposed using tetramethylammonium-functionalized polysulfone as an AEM. The nanophase separation between hydrophilic and hydrophobic regions in quaternized polysulfone allows for the formation of effective continuous pathways for OH^−^ conduction pathways, with hydroxide conductivity reaching 77.5 mS/cm at 80 °C. Furthermore, the use of relatively thin membranes can also enhance the performance of AEMWE [26], and a 28 μm-thick AEM demonstrated hydrogen barrier performance comparable to that of a 175 μm-thick PEM [28], proving the feasibility of this strategy.

**Figure 2:**
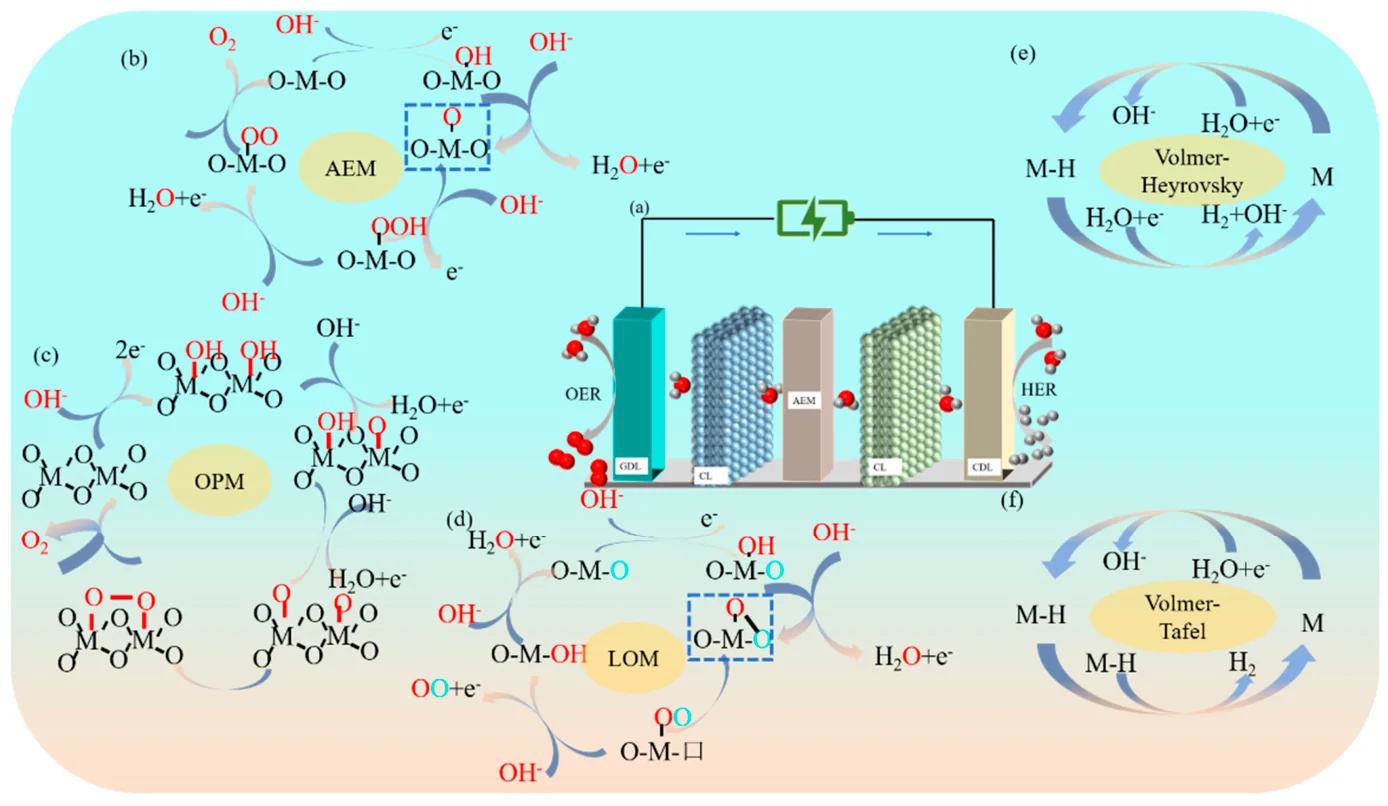
Schematic diagram of anion exchange membrane water electrolysis.

This is a schematic diagram of the electrocatalytic mechanism of water electrolysis (as shown in Figure 2a–f), illustrating three pathways for the oxygen evolution reaction (OER): AEM, OPM, and LOM, as well as two pathways for the hydrogen evolution reaction (HER): Volmer-Heyrovsky and Volmer-Tafel. It presents the intermediate states and electron transfer processes of the reactions.

**1.**  **Three detailed mechanisms of oxygen evolution reaction (OER)**

Adsorption Evolution Mechanism (AEM): This is the most classic OER pathway, where the core process involves the adsorption of reactants on the catalyst surface, gradual dehydrogenation, and ultimately the coupling to generate oxygen: Step 1: Water molecules adsorb on the catalyst surface and lose one electron, forming O–M–OH; The O–M–OH (metal-hydroxyl) intermediate state is formed, releasing OH^−^ simultaneously; the second step: O–M–OH undergoes further dehydrogenation to generate the O–M=O (metal-peroxy) intermediate state, releasing OH^−^ again and transferring electrons; the third step: two O–M=O intermediate states undergo oxygen-oxygen coupling to produce O_2_, while the active sites on the catalyst surface are restored, completing the catalytic cycle. The rate-determining step of this pathway is usually the dehydrogenation process in the second step, and the electronic structure of the catalyst directly affects the energy barrier of this step. The Oxygen Participation Mechanism (OPM) differs from the Adsorption Energy Mechanism (AEM) in that it relies solely on surface adsorbed species. OPM directly utilizes oxygen atoms in the catalyst lattice: Step 1: Water molecules adsorb on the catalyst surface and react with lattice oxygen to form an O–M–O–M (bridged oxygen) intermediate state, releasing OH^−^ simultaneously; Step 2: The bridged oxygen intermediate state undergoes reconstruction, with lattice oxygen participating in the reaction to generate O_2_, and oxygen vacancies are formed on the catalyst surface; Step 3: Water molecules fill the oxygen vacancies, restoring the catalyst lattice structure and completing the cycle. The advantage of OPM is that it can lower the energy barrier of the rate-determining step, making it particularly suitable for highly active transition metal oxide catalysts (such as IrO_2_ and RuO_2_). Lattice Oxygen Mechanism (LOM): This is an extension path of OPM, with the core being the direct oxidation of lattice oxygen. The first step involves the adsorption of water molecules on the catalyst surface, forming an O–M–OH intermediate state with lattice oxygen, releasing OH^−^; the second step involves the oxidation of lattice oxygen in O–M–OH, generating O_2_, while oxygen vacancies are formed on the catalyst surface; the third step involves water molecules filling the oxygen vacancies, completing the catalytic cycle. The reaction energy barrier of LOM is usually lower, but it requires higher lattice stability of the catalyst to avoid structural collapse of the catalyst caused by oxygen vacancies [49,50,51,52,53,54,55].

**2.**  **Two detailed mechanisms of hydrogen evolution reaction (HER)**

Volmer-Heyrovsky mechanism is the mainstream pathway for HER under alkaline conditions, involving two key steps: adsorption and desorption. Volmer step: Water molecules adsorb on the catalyst surface and acquire electrons, generating an M–H (metal-hydrogen) adsorption state while releasing OH^−^; Heyrovsky step: The M–H adsorption state reacts with another water molecule, acquiring electrons to form H_2_, while releasing OH^−^, and the catalyst surface recovers active sites. The rate-determining step of this pathway is usually the Heyrovsky step, and the hydrogen adsorption energy of the catalyst directly affects the reaction rate. Volmer-Tafel mechanism: This is the typical pathway for HER under acidic conditions, with the core being the bimolecular recombination of hydrogen atoms [56,57,58,59,60,61,73]:

Volmer step: Consistent with the above, water molecules adsorb to form M–H adsorbed states; Tafel step: Two M–H adsorbed states undergo recombination on the catalyst surface, directly generating H_2_ and releasing two OH^−^. The rate-determining step of this pathway is the Tafel step, which requires suitable hydrogen adsorption sites on the catalyst surface, commonly found in noble metal catalysts such as Pt and Pd. The practical significance of the reaction mechanism is as follows: Catalyst design: By regulating the electronic structure, surface defects, or lattice oxygen content of the catalyst, the energy barrier of the rate-determining step can be optimized, enhancing catalytic activity. For example, designing transition metal oxides with oxygen vacancies can promote the OPM pathway; Reaction condition optimization: Under different pH conditions, the dominant pathway of HER will switch, and the active sites of the catalyst can be matched by adjusting the electrolyte pH; Performance evaluation: By comparing the reaction energy barriers of different mechanisms, the activity differences of catalysts can be explained, guiding the construction of an efficient water electrolysis system [58,59,60,61,62,63,64,65].

#### 2.1.2. Strategies for Improving AEM Stability

To improve stability, existing research has primarily focused on the following two approaches:

(1) Designing ether-free main chains from the perspectives of monomer structure and polymerization reactions. In AEM materials, the aromatic ether bonds on the polymer main chain degrade more rapidly in alkaline environments due to the presence of nearby electron-withdrawing groups (such as quaternary ammonium salts), leading to degradation of the membrane main chain; in contrast, all-carbon-based polymers without aromatic ether bonds exhibit good long-term alkali stability [32]. Therefore, developing polymer membrane materials without aryl ether bonds is an effective approach to improving the alkali stability of AEMs. Yang et al. [42] prepared a non-aryl ether piperidine-based anion exchange membrane, QMter-co-Mpi, featuring a twisted molecular structure; durability testing results showed that its stability exceeds 500 h. Xu et al. [4] synthesized a stable, ether-free polymer with a pyridine side chain by modifying the side chains with varying ratios of hydrophilic (2-bromoethyl)trimethylbromide and hydrophobic bromohexyl groups. The ether-free alkyl backbone and steric shielding conferred enhanced resistance to nucleophilic attack on the membrane. Consequently, after soaking the BTAB_0.8_-HB_0.2_/PBP membrane in a 1 mol/L KOH solution at 80 °C for 1008 h, it still retained 85% of its initial conductivity.
(2) Developing new cationic groups with high chemical stability. Earlier studies primarily used trimethylammonium salts as functional groups; however, research has shown that under high-temperature, alkaline conditions, these quaternary ammonium salts are prone to nucleophilic attack by OH^−^, leading to substitution or *β*-elimination reactions, which in turn cause the AEM to degrade and lose its ability to transfer OH^−^ [12]. To enhance the stability of quaternary ammonium cationic functional groups and replace traditional trimethylammonium salts, research has focused on various chemically stable functional groups, including aromatic quaternary ammonium salts, non-aromatic cyclic amine salts, and metal-centered cations [11,32]. Research has shown that [33] grafted a sterically hindered imidazolium cation with different substituents at positions C_2_ and N_3_ onto polyaryletherketone; the resulting PAEK-APMBI membrane remained stable after four weeks of immersion in a 10% KOH solution at 60 °C. Hu et al. [43] linked butyl sulfonate side chains of varying concentrations to the [2,2′-(1,4-naphthalene)-5,5′-benzimidazole] polymer backbone to obtain NPBI-BS-X membranes. Among these, the NPBI-BS-47 membrane retained 89% of its initial conductivity after 1000 h of non-in situ alkali stability testing in 8 mol/L KOH.

### 2.2. OER Electrocatalysts

#### 2.2.1. OER Mechanism

**Figure 3:**
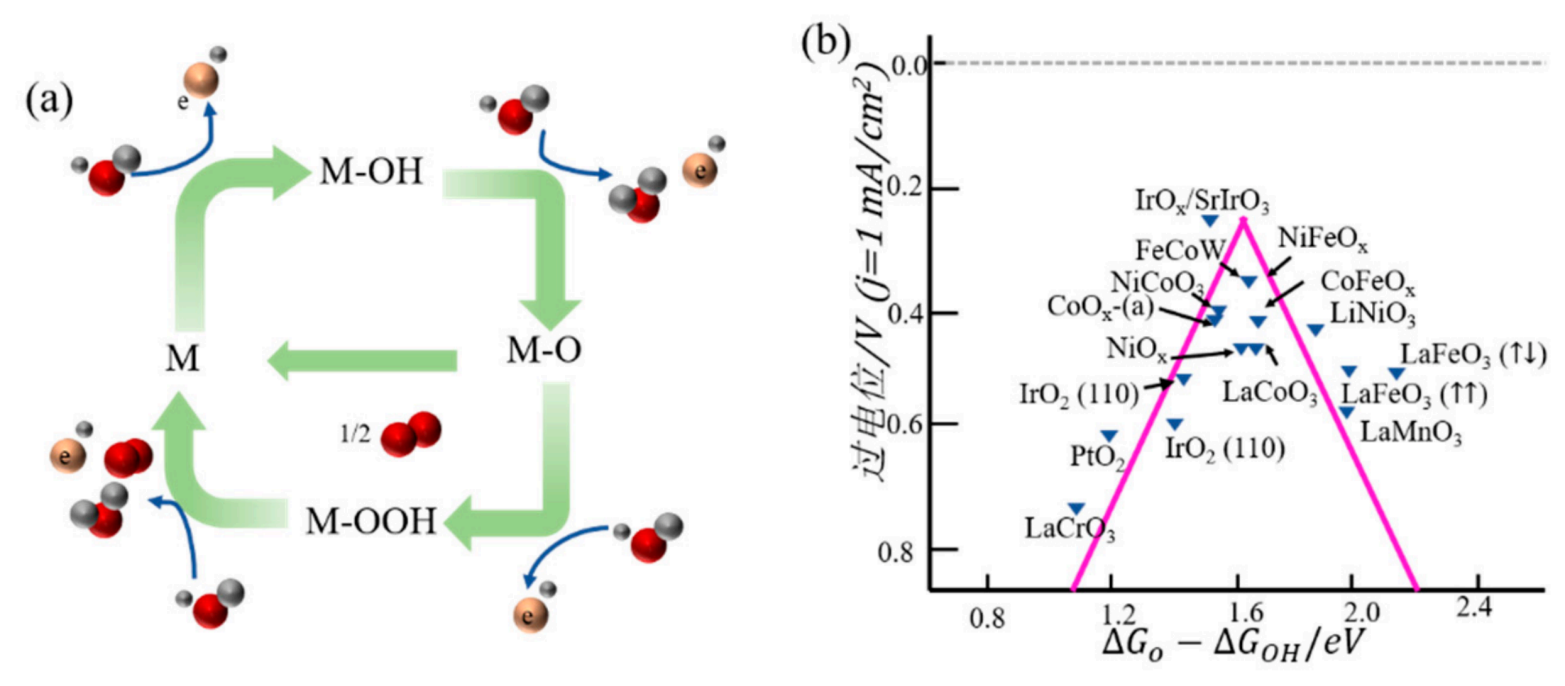
(**a**) Alkaline OER mechanism [31]; (**b**) metal oxide OER volcano plots [42].

The mechanism of OER in both alkaline and acidic media has not yet been fully elucidated. Among the proposed OER mechanisms, the four-step mechanism is the widely accepted mechanism for alkaline OER, as shown in Figure 3a. The specific reaction steps are as follows [31]:

$$M+{OH}^{-}\to MOH+e^{-}$$

$$MOH+OH^{-}\to MO+H_{2}O+e^{-}$$

$$MO+OH^{-}\to MOOH+e^{-}$$

$$MOOH+OH^{-}\to M+O_{2}+H_{2}O+e^{-}$$

where M represents an adsorption site on the metal surface. In the first step, MOH is formed through the single-electron oxidation of hydroxide ions adsorbed at the active sites; in the second step, MOH is converted to MO, and two protons and an electron are removed; in the third step, MO combines with hydroxide ions to form MOOH through single-electron oxidation; finally, MOOH reacts with hydroxide ions to produce O_2_ and water, and the M active site returns to its initial state [30].

Although OER is favored under alkaline conditions, its energy requirements remain higher than those of HER due to the complex four-step reaction pathway. Based on the binding energy relationships in the OER mechanism, researchers have constructed OER volcano plots for various metal oxide surfaces, as shown in Figure 3b. Among these, IrO*_x_* remains the best OER catalyst to date and serves as the benchmark for transition-metal-based OER catalysts. However, the relatively slow OER kinetics resulting from the four-step reaction remain an unresolved issue. Furthermore, due to the requirement for continuous adsorption and desorption of reaction intermediates, research efforts have focused on identifying variable-valent species and optimizing the adsorption energies of intermediates to develop highly active OER catalysts.

#### 2.2.2. Recent Advances in OER Electrocatalyst Research

To fully leverage the low-cost advantage of AEMWE, the development of non-precious metal catalysts is crucial for reducing the cost of AEMWE. In recent years, extensive research has been conducted on transition metal catalysts for OER under weakly alkaline conditions. These materials primarily focus on first-row transition metals, such as nickel (Ni), cobalt (Co), iron (Fe), and copper (Cu), as well as the second-row transition metal molybdenum (Mo). Among these, Ni-based OER catalysts exhibit good activity and stability under alkaline conditions and are widely used as OER electrocatalysts. Due to its high oxygen affinity, Fe tends to adsorb OH^−^ and is typically employed as a kinetics promoter for OER [34,66,67,68,69,70]. Extensive research indicates that NiFe-based electrocatalysts possess high intrinsic activity and a low intermediate adsorption energy barrier, offering broad application prospects in weakly alkaline water electrolysis OER. Furthermore, due to its lower cost and oxidation resistance in the oxidation reaction zone [37], Co is also used as a substitute for precious metals, similar to Ni-based OER electrocatalysts. Similar to NiFe-based catalysts that exhibit excellent OER performance, the introduction of Fe together with Co to form CoFe-based catalysts also demonstrates good OER activity. Based on the above analysis of metal elements, current research on non-precious metal-based OER electrocatalysts is categorized into the following four aspects according to their composition.

First, transition metal oxides. Non-precious metal-based transition metal oxides are widely used as OER electrocatalysts due to their low cost, abundant raw materials, high activity, and stable performance. Their overpotential can be reduced by optimizing particle size and surface area, as well as by adjusting the structure, such as by adopting spinel and perovskite structures. Seh, Z.W et al. [44] used NiFe_2_O_4_ prepared via a liquid phase method as an oxygen evolution catalyst in an AEM electrolyzer. They deposited the nickel ferrite onto a Fumasep@FAA3-50 anion exchange membrane using spray coating technology to create a catalyst-coated membrane. In single-cell testing, at 60 °C and 2.2 V, the current density reached 3 A/cm^2^, exceeding that of commercial catalysts using IrO_2_.

The second category includes alloy electrocatalysts. Multicomponent alloys can overcome the high overpotential generated by single non-precious metal electrocatalysts; by alloying and modifying the surface electronic structure, activity can be maximized. Research in [45] showed the synthesizing of a nitrogen-doped carbon-coated CoFe electrocatalyst in three steps via hydrothermal reaction, in situ polymerization of dopamine, and carbonization. The CoFe/NC catalyst exhibited excellent catalytic activity for OER, requiring only a 340 mV overpotential at a current density of 10 mA/cm^2^ in 1.0 mol/L KOH. Chen et al. [11] developed an AEM electrolyzer using a poly(fluorenyl-arylpiperidine) membrane and a NiFe-based catalyst. At a voltage of 2.0 V, the current density reached 1.62 A/cm^2^, and the system operated stably for over 1000 h at 60 °C and a current density of 0.5 A/cm^2^, demonstrating good electrolytic performance and stability. The third category includes hydroxide electrocatalysts. Due to their low cost, high specific surface area, and unique electron distribution, they are considered one of the best electrocatalysts for the oxygen evolution reaction (OER). Typical electrocatalysts for OER include Ni(OH)_2_, Co(OH)_2_, NiFe-LDH, and NiFeOOH. Li et al. [53] induced lattice distortion in NiFe-LDH nanosheets through cerium doping to prepare the NiFeCe-LDH@CP catalyst, which features a significantly twisted lattice and defects. The lattice distortion increased the number of active sites, and the catalyst exhibited excellent OER activity, with an overpotential of only 232 mV and a Tafel slope of 31.69 mV/dec at 10 mA/cm^2^, outperforming traditional IrO_2_ catalysts. The fourth category includes transition metal phosphides. Compared to other materials, phosphides readily form a variety of structures and exhibit unique physicochemical properties, particularly strong electrical conductivity and exceptional corrosion resistance. Sankar et al. [46] developed a self-supporting metal phosphide catalyst, Ni_2_P-Fe/NF, based on commercially available porous 3D foam nickel, and used it as the anode in an electrode system, demonstrating high OER performance (1 A/cm^2^@1.73 V). This result indicates that the incorporation of P can accelerate the OER kinetic characteristics. In summary, the currently much-discussed NiFe catalysts (layered double hydroxides/oxides) and Ni-based or Cu/Co-mixed spinel-type and perovskite-type oxides remain the most promising non-precious metal-based catalysts in AEMWE. Furthermore, further research into the OER mechanism is crucial for the development of high-performance non-precious metal-based OER catalysts from a mechanistic perspective. Currently, research aimed at enhancing catalyst performance at the mechanistic level primarily focuses on two aspects: surface restructuring and lattice oxidation mechanisms. Surface restructuring can lead to a more active surface structure, which is beneficial for enhancing the activity of the OER process [47]. Wang et al. [47] proposed a cation redox-regulated method to modulate the in situ leaching of the catalyst and redirect the dynamic surface restructuring of layered LiCoO_2-*x*_Cl*_x_*. Chlorine doping reduces the likelihood of in situ cobalt oxidation and lithium leaching, thereby inducing the surface of LiCoO_1.8_Cl_0.2_ to transform into a self-terminating amorphous hydroxide phase during the OER process. The performance of the surface-reorganized LiCoO_1.8_Cl_0.2_ surpasses that of many state-of-the-art OER catalysts and exhibits excellent stability. The lattice oxidation mechanism is not affected by the ratio of adsorption energies between OH* and OOH*. Since breaking this linear relationship offers the potential for achieving high activity, more efficient OER electrocatalysts can be designed based on the lattice oxidation mechanism [46,47,48,49,50].

### 2.3. HER Electrocatalysts

#### 2.3.1. HER Mechanism

**Figure 4:**
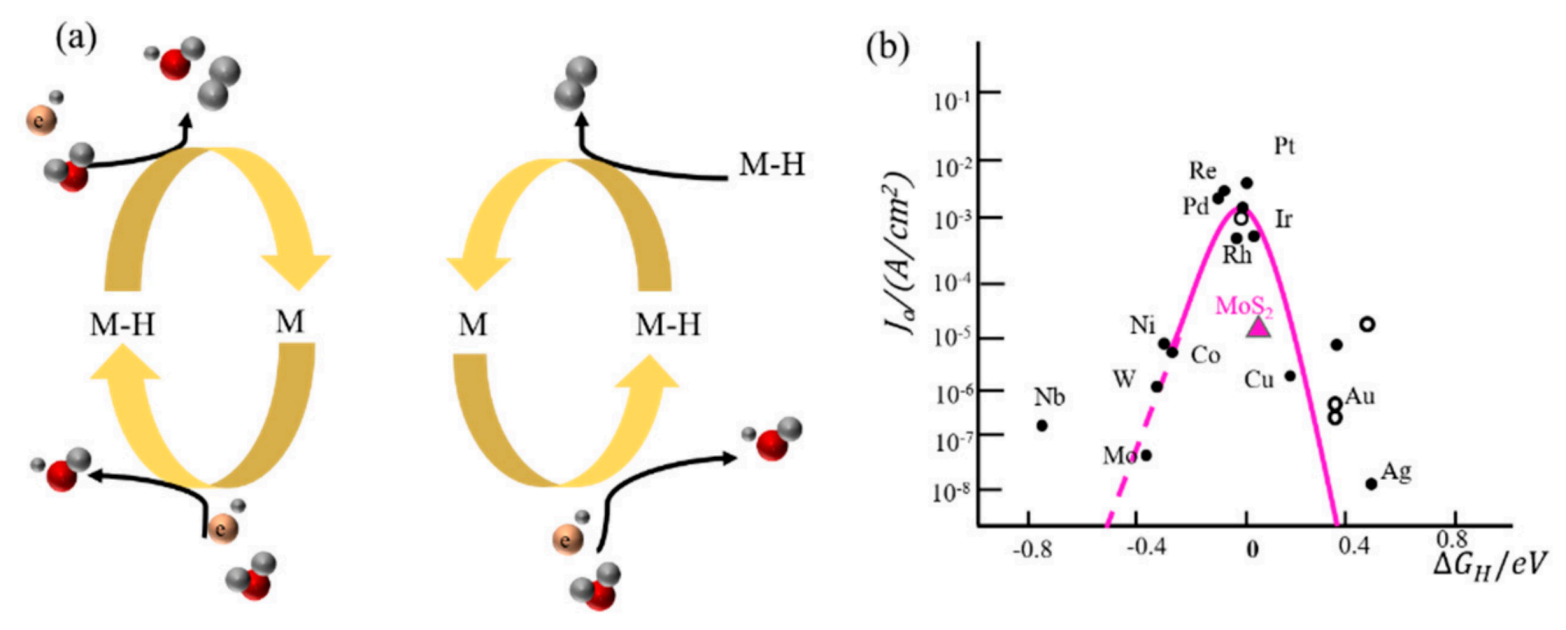
(**a**) Alkaline HER mechanism [41]; (**b**) monometallic HER volcanic curve [49].

The hydrogen evolution reaction (HER) in an acidic environment is a two-electron catalytic process with relatively fast kinetics. However, in a basic environment, HER proceeds relatively slowly because water dissociation occurs at the cathode. HER in a basic environment typically follows the three-step mechanism proposed by Volmer, Heyrovsky, and Tafel [32], as shown in Figure 4a:

$$\mathrm{Volmer}:\;H_{2}O+M+e^{-}\to MH+OH^{-}$$

$$\mathrm{Heyrovsky}:\;H_{2}O+MH+e^{-}\to M+OH^{-}+H_{2}$$

$$\mathrm{Tafel}:\;2MH\to 2M+H_{2}$$

The Volmer reaction involves the electrochemical reduction of water to hydrogen. Compared to the HER under acidic conditions, the Volmer reaction under alkaline conditions requires the dissociation of water molecules and thus demands more energy. Subsequently, hydrogen molecules can be formed via two pathways: the Heyrovsky step, which involves electrochemical hydrogen evolution, and the Tafel step, which involves chemical hydrogen evolution. Therefore, to achieve high catalytic activity in HER, the binding strength between the catalyst and hydrogen must be moderate—neither too strong nor too weak. Studies have shown that as the catalyst’s ΔGH approaches 0, HER activity increases [47,65,66,67,68,69,70,71,72,73]. The HER volcano plots for single-metal systems are shown in Figure 4b. Among these, Pt/C is a highly efficient HER noble metal catalyst and serves as a benchmark for evaluating the performance of transition metal catalysts.

#### 2.3.2. Recent Advances in HER Electrocatalyst Research

In recent years, active research has continued into the development of transition-metal-based HER electrocatalysts under weakly basic conditions. Research on HER electrocatalysts under weakly basic conditions has primarily focused on non-precious metals such as nickel (Ni), cobalt (Co), molybdenum (Mo), copper (Cu), and iron (Fe). These non-precious metal elements are structurally engineered, through element doping, alloying, phosphide and sulfide formation, or the introduction of carbon allotropes such as carbon nanotubes and graphene, to create nanomaterials featuring defect sites and high specific surface areas. Current research on non-precious metal-based HER electrocatalysts is primarily divided into the following two categories based on the metal element. The first is Ni-based electrocatalysts. Due to their high activity and excellent corrosion resistance in alkaline media, as well as greater stability compared to other transition metals (such as Fe and Co), Ni-based catalysts are being used as HER catalysts to replace Pt-based catalysts [49]. For example, in NiFe-based OER catalysts, Co enhances the active surface area and catalytic activity of Ni [50,51,52,53,54,55], and the synergistic effect of the Ni–Co interaction reduces the energy barrier for H–OH bond dissociation, accelerating the Volmer step [51]. Consequently, NiCo-based catalysts are frequently used as HER catalysts. Research has shown that [52] synthesized ternary NiCoP nanostructured HER electrocatalysts via electrochemical deposition at room temperature; their HER performance surpassed that of binary alloys, with the NiCoP-I electrocatalyst exhibiting a low Tafel slope of 53 mV/dec in alkaline solution at a current density of 10 mA/cm^2^. Furthermore, the presence of Mo can promote the Volmer step and enhance intrinsic activity [54]; therefore, NiMo-based alloys are also commonly used as alkaline HER electrocatalysts. Wang et al. [66] constructed a low-cost, precious-metal-free electrolyzer using an AEM and plasma-sprayed electrodes. They developed and tested several electrode materials under conditions of 60 °C and a 1 mol/L KOH electrolyte. The AEM electrolyzer using a NiAlMo electrode achieved a potential of 2.086 V at a current density of 2 A/cm^2^, a performance comparable to that of industrial megawatt-scale PEM electrolyzers.

Second, there are Co-based electrocatalysts. Co has attracted attention for its role in improving the kinetics of nickel-based catalysts. Unlike Co-based OER electrocatalysts containing CoFe and Co oxides, the Co-based HER catalysts used as cathodes in AEMWE are typically Co phosphides or sulfides, as heteroatoms such as heteroatoms such as phosphorus, sulfur, and nitrogen can serve as active sites participating in the HER [56]. Li et al. [53] prepared a Co_2_P/Ni_2_P nano-hybrid HER electrocatalyst with abundant nanopores via a two-step thermal phosphorization process; in a 1 mol/L KOH electrolyte, the overpotential at 10 mA/cm^2^ was as low as 51 mV. J. Zhao and Y. Guo et al. [51] prepared Co_2_PNi_12_P_5_/NF||Fe_2_P-Ni_12_P_5_/NF heterojunction on foam nickel, which exhibited good HER performance and similarly demonstrated good performance when assembled into a two-electrode system simulating industrial electrolysis (1 A/cm^2^@1.678 V). Moreover, when applied in an AEM electrolyzer, it outperformed commercial Pt/C/NF||IrO_2_/NF catalysts, providing insights for the development of large-scale hydrogen production electrocatalysts for industrial use.

### 2.4. Bifunctional Catalysts

Bifunctional catalysts must exhibit both catalytic activity and durability for both HER and OER. In PEM electrolysis, Pt/C for HER and RuO_2_/IrO_2_ for OER have not demonstrated the ability to catalyze both reactions simultaneously; however, transition-metal-based catalysts have exhibited this property. Using the same material combination for both the anode and cathode not only has the potential to reduce the manufacturing cost of the electrolyzer but also simplifies the system. In recent years, the bifunctionality of Ni-based and Co-based materials has been extensively studied. Among these materials, NiFe and NiCo-based alloys, sulfides, phosphides, oxides, and hybrid compounds exhibit low overpotentials, low Tafel slopes, and good HER and OER stability under weakly alkaline conditions. However, the HER kinetic performance of these catalysts remains inferior to that of Pt/C, while some catalysts exhibit better OER kinetic performance than IrO_2_/RuO_2_. In summary, bimetallic catalysts based on transition metals have demonstrated good performance in AEMWE and hold research potential. The NiFe_2_O_4_@ N/rGO bifunctional catalyst exhibited excellent OER kinetic performance in 1 mol/L KOH, with an overpotential of 252 mV at 20 mA/cm^2^. The higher conductivity, specific surface area, and oxygen vacancy defects in N/rGO resulted in superior OER catalytic performance compared to IrO_2_. For HER, the overpotential was higher than that of Pt/C, approximately 157 mV at 10 mA/cm^2^. When used as a bifunctional electrode in an AEMWE, it exhibited low cell voltages of 1.60 V and 1.67 V at current densities of 10 mA/cm^2^ and 20 mA/cm^2^, respectively. Pan et al. [54] used a NiCoP@FeP bifunctional catalyst prepared by the phosphating method in an AEMWE, achieving an overpotential as low as 1.58 V in a 1 mol/L KOH electrolyte at a current density of 10 mA/cm^2^. Lee et al. [50] successfully synthesized core–shell structured nickel–cobalt alloy–nickel–cobalt phosphide NiCo@ NiCoP/NF nanorods, which were used as a bifunctional catalyst in an electrolytic cell. At a current density of 10 mA/cm^2^, the overpotential was as low as 1.58 V, and the catalyst exhibited electrochemical durability exceeding 100 h.

### 2.5. Anion Exchange Ionomers

Anion exchange ionomers (AEIs) are essentially polymer matrices with a structure similar to that of AEMs [68,69,70,71,72]. They act as a binder between the catalyst particles, the GDL carrier, and the AEM, facilitating additional ion transport between the catalyst’s active sites and the ion exchange membrane, thereby helping enhance electrocatalytic activity. Anion exchange ionomers are typically mixed with the catalyst to form an “ink” which is then applied to the substrate/electrode surface during MEA fabrication using the catalyst-coated substrate (CCS) method, or applied to the membrane using the catalyst-coated membrane (CCM) method. Similar to research on AEMs, commercial AEIs with high thermal and chemical stability, such as perfluorosulfonic acid-based AEIs, are available for PEMWE. However, no standard AEIs currently exist for AEMWE; traditional AEIs commonly used for AEMWE include quaternary amine polysulfone [59,60,61]. Therefore, the development of AEIs with high ionic conductivity and stability is a key focus of AEMWE technology. As a key component of the membrane electrode, a high-quality ionomer can significantly enhance AEMWE performance. The ionomer should possess the following characteristics: first, high ionic conductivity, rapid water diffusion capability, and rapid gas permeability; and second, good stability, including oxidative stability, thermal stability, durability, and mechanical stability.

The loading and distribution of the copolymer relative to the catalyst have a significant impact on electrolytic performance. Vincent et al. [59] investigated the effect of copolymer loading (9–33%) on the electrolytic performance of the catalyst; the results indicated that the lowest loading yielded the best performance. Park et al. [60] achieved optimal MEA performance with a 20% mass fraction of the ionomer. If the ionomer loading is too low, it cannot facilitate charge transfer and mass transport; conversely, excessively high ionomer loading can obstruct the catalyst’s active sites. Studies have shown that the commercially available Aemion copolymer is incompatible with the nickel-based anode catalyst in AEMWE [64]. Therefore, it is necessary to select an appropriate copolymer based on the specific catalyst and optimize the ratio of copolymer to catalyst loading at the electrode to achieve optimal electrocatalytic performance in the electrolyzer.

In electrolysis, the AEI plays a more significant role than the AEM [27]. This is because there are minimal performance differences among the various polymer architectures used in AEMs, and the performance of AEMWE primarily depends on the catalytic activity of the HER and OER, which is largely influenced by the interactions between the AEI and the catalyst. Research by Chen et al. [64] indicates that the development of ion–polymer composites is a key approach to maximizing the lifespan of AEMWE systems. Compared to AEM, reduced swelling of the ion–polymer composite in the catalyst layer extends the lifespan of AEMWE systems operating under pure water conditions by a factor of four. Therefore, there is an urgent need to develop high-performance ion–polymer composites for AEMWE.

### 2.6. Membrane Electrode Assembly

**Figure 5:**
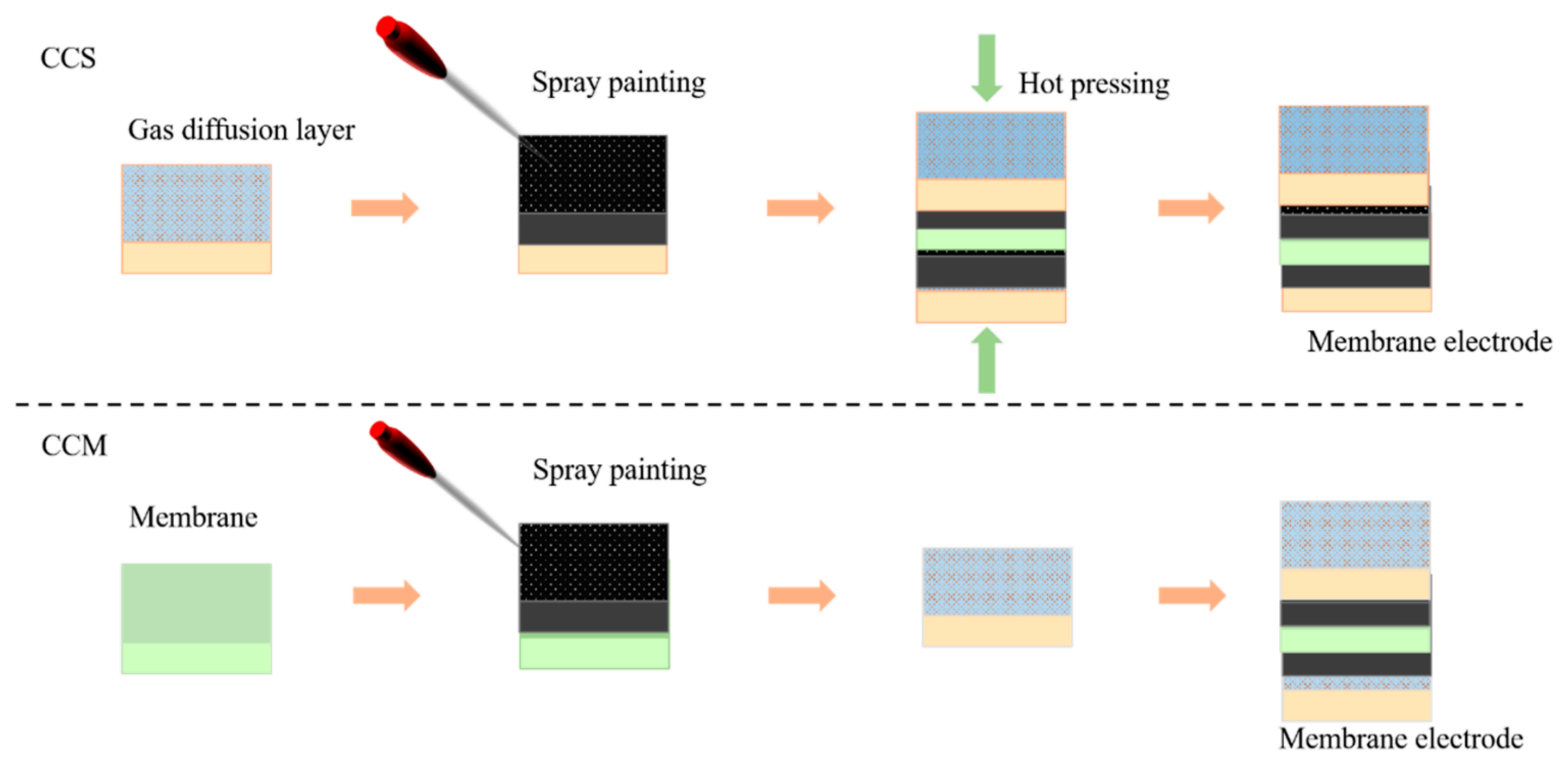
Summarized of CCS and CCM syntheses routes [71].

The membrane electrode assembly (MEA) is the core component of an AEM electrolyzer, primarily consisting of the AEM, the interlayer, the anode and cathode catalyst layers, and the porous transport layer. The AEM, anode, cathode electrocatalysts, and interlayer have been discussed previously and will not be repeated here. The MEA provides a critical interface for electrochemical reactions and mass transfer, directly determining the performance and durability of alkaline water electrolysis. Therefore, the development of high-performance, low-cost MEAs is key to driving the large-scale application of AEMWE. There are two methods for preparing MEAs: CCS and CCM, as shown in Figure 5.

In the CCM method, a mixture of electrocatalyst and polymer is prepared as a slurry, which is then applied to both sides of the AEM using methods such as spraying or spin coating and dried; it is then placed between gas diffusion layers and assembled via mechanical or hot pressing. In the CCS method, the electrocatalyst slurry is directly deposited onto the gas diffusion layer and then sintered to form the electrode; the AEM is encapsulated between the gas diffusion layer or the electrode to form the membrane electrode assembly. The CCS method is relatively easy to implement and suitable for large-scale production; however, the direct deposition of the catalyst onto the gas diffusion layer prevents sufficient contact between the membrane and the catalyst layer, thereby affecting catalyst activity. A MEA prepared using the CCM method exhibits higher catalyst utilization and electrochemical reaction rates compared to a CCS-based MEA [66]. However, the manufacturing process for CCM is relatively complex, and for membranes thinner than 20 μm, some of the solvent in the catalyst ink may dissolve the membrane. Additionally, membrane creep behavior in CCM can reduce the membrane’s durability [68]. Given the use of non-precious metal-based electrocatalysts, further research is needed to compare CCS-based MEAs with CCM-based MEAs in order to identify the most suitable MEA fabrication method and compatible non-precious metal-based materials.

In recent years, self-supporting catalytic electrodes based on surface-modified porous supports have attracted significant attention. Self-supporting catalytic layers consist of modified or reinforced porous supports and typically do not require polymers (unlike CCS), meaning that the catalytic active material grows directly in situ on a conductive substrate. Compared to traditional powdered catalysts, self-supporting electrodes avoid the use of polymer binders, thereby preventing the inhibition of bubble diffusion and the exposure of active sites [68]. This tight anchoring effectively enhances the mechanical stability of self-supporting electrodes and ensures efficient electron transfer between the active material and the substrate. Consequently, self-supporting electrodes are more suitable for alkaline water electrolysis under high current densities and long-term operation [69]. Currently, the primary methods for preparing self-supporting electrodes are electrodeposition and hydrothermal/solvothermal methods. Recently, Wan et al. [68] designed a novel 3D-ordered MEA based on a highly porous catalytic layer (CL), a membranous layer (ML), and a 3D CL/ML interface structure. This structure features abundant active sites and efficient mass transfer pathways; when applied to AEMWE, it demonstrated excellent electrochemical performance (3.1 A/cm^2^@2 V). This approach to constructing a 3D-ordered MEA between the CL and ML provides new insights for the design of high-performance AEMWE membrane electrodes.

### 2.7. Porous Transport Layer and Bipolar Plate

The porous transport layer (PTL), also known as the gas/liquid diffusion layer or current collector, is located between the bipolar plate and the catalyst layer. Its primary functions are to facilitate electrolyte and gas transport, as well as the conduction of electrons and heat. In PEM electrolyzers, due to the harsh environment on the anode side—characterized by high potential, oxygen-rich conditions, and strong acidity—expensive titanium-based PTL materials are commonly used. In contrast, in AEM electrolyzers, due to the weakly alkaline conditions, inexpensive foamed nickel is typically used for the anode PTL, while the cathode may use either foamed nickel or carbon cloth, significantly reducing costs. PTL plays a critical role in the performance of AEMWE [71]; therefore, research into PTL with high electrical and thermal conductivity, strong gas-repellent/hydrophilic properties, good mechanical characteristics, and high corrosion resistance is of paramount importance. Current research primarily focuses on optimizing PTL morphology and porosity, as well as developing new materials. Additionally, by studying heat and mass transfer within the electrolyzer, thermal management can be optimized to further improve electrolysis performance. Research has shown that [71,72] used simulation models to investigate the effects of membrane and PTL thermal conductivities on temperature gradients in AEM electrolyzers. Their findings revealed significant discrepancies between the core temperature of the cell and the measured temperature, highlighting the limitations of relying solely on electrolyte control to regulate reaction temperatures.

Bipolar plates are multifunctional components in aqueous electrolysis cells. Their primary functions include connecting adjacent cells within the stack, ensuring the transport of charge carriers between adjacent cells, supplying reactants (H_2_O) and removing gaseous products (H_2_ and O_2_), and providing mechanical support for current collectors, membranes, and electrodes. In addition, bipolar plates play a crucial role in heat transfer and water management [75]. In PEM electrolyzers, due to the harsh, highly acidic environment, bipolar plates (BPs) still require the use of expensive titanium plates to ensure thermal conductivity, electrical conductivity, corrosion resistance, and chemical stability. In AEMWE, however, BPs—which currently largely inherit AWE technology—are typically made of nickel-plated stainless steel [73]. It is worth noting that bipolar plates account for 50% of the total cost of an electrolyzer [74]. With the development of pure aqueous AEMWE technology, it is possible to design lower cost bipolar plates to further reduce the overall cost of AEM electrolyzers and enhance their competitive advantage. Furthermore, as the interface for electrolyte and gas transport, research on heat and mass transfer in bipolar plates is of great importance. In PEMWE, studies have explored the rational design of various flow channel structures, such as parallel flow fields, single serpentine flow fields, and multi-serpentine flow fields [76]. Other studies have used flow channel modeling to analyze the effects of channel height, blockage, ridge width, and flow channel boundaries [77], with the aim of identifying optimal flow channel structures and parameters to minimize flow resistance, promptly discharge hydrogen and oxygen generated by the electrochemical reaction, reduce mass transfer resistance, and lower voltage drop. These studies provide insights for the design of high-efficiency bipolar plates in AEMWEs.

### 2.8. Electrolyte

The electrolyte plays a crucial role in enhancing the electrolysis efficiency of AEMWE. Commonly used electrolytes in AEMWE include KOH, K_2_CO_3_, and pure water. Unlike the high-concentration alkaline electrolytes used in traditional AWEs, AEMWEs can utilize low-concentration alkaline solutions because AEMs can conduct OH^−^. This avoids safety issues caused by highly corrosive electrolytes during experiments, not only reducing electrolysis costs but also enhancing system flexibility. However, lower concentration alkaline solutions undoubtedly affect ion transport and reduce electrolysis performance. Generally, electrolytic performance improves as electrolyte concentration increases, but this improvement is limited. When the KOH concentration in the electrolyte solution was increased from 0.1 mol/L to 0.3 mol/L, cell performance improved; however, when the concentration was further increased from 0.3 mol/L to 1.0 mol/L, the improvement in performance was negligible [75]. This is primarily because a slight increase in electrolyte concentration helps accelerate the kinetic characteristics of OER and HER, reduce the membrane’s ohmic resistance, and increase ion mobility. However, further increasing the electrolyte concentration leads to an increase in solution viscosity and blocks catalytic active sites due to the formation of bubbles on the surface. Currently, commonly used weakly alkaline solutions are KOH and K_2_CO_3_; while the kinetics of carbonates are generally lower than those of hydroxides, there remains controversy regarding the selection between the two. Kiessling et al. [67] compared the electrolytic performance of electrolytes with the same conductivity and pH. In the kinetic current region, the electrolytic performance of the three electrolytes followed the trend of 0.5 mol/L KOH > 18 mmol/L KOH > 0.82 mol/L K_2_CO_3_; however, at current densities of 700–2000 mA/cm^2^, the trend shifts to 0.5 mol/L KOH > 0.82 mol/L K_2_CO_3_ > 18 mmol/L KOH, indicating that carbonates may be the superior choice under steady-state and high-current-density operating conditions.

To enable large-scale hydrogen production and avoid costly water pretreatment, electrolyzer technology must be compatible with water as it occurs naturally. Seawater is abundant and, in principle, enables more efficient water electrolysis (seawater resistivity 20 Ω·cm << pure water resistivity 18 MΩ·cm), making it an ideal electrolyte. However, Cl^−^ and microorganisms in seawater corrode metals; in particular, Cl^−^ triggers the chlorine evolution reaction, which severely hinders the oxygen evolution reaction (OER) and poses a significant challenge to OER electrodes [66]. Furthermore, Ca^2+^ and Mg^2+^ present in seawater deposit on the electrode surface and within pores during electrolysis, blocking catalytic active sites [73.77] and severely impairing catalytic activity. Developing a seawater electrolyzer with high electrochemical activity and durability is a daunting task. Among numerous catalysts, NiFe layered double hydroxide (LDH) electrocatalysts exhibit high OER activity in seawater [74]. The NiFe-LDH catalyst-based AEM seawater electrolyzer developed by Xing et al. [52] operated for over 1000 h at 300 mA/cm^2^ and demonstrated excellent electrolytic performance. In recent years, domestic researchers have achieved breakthrough progress in seawater electrolysis for hydrogen production. The strategy for in situ direct seawater electrolysis to produce hydrogen without desalination, developed by the team led by Academician Xie Heping [70], has been successfully applied to the world’s first offshore wind power seawater hydrogen production demonstration project, breaking through a half-century-old challenge in the field of seawater electrolysis for hydrogen production. This will undoubtedly accelerate the industrialization of large-scale renewable energy hydrogen production globally.

## 3. Working Principle and Performance Requirements of Anion Exchange Membrane

### 3.1. Working Principle of Anion Exchange Membrane

**Figure 6:**
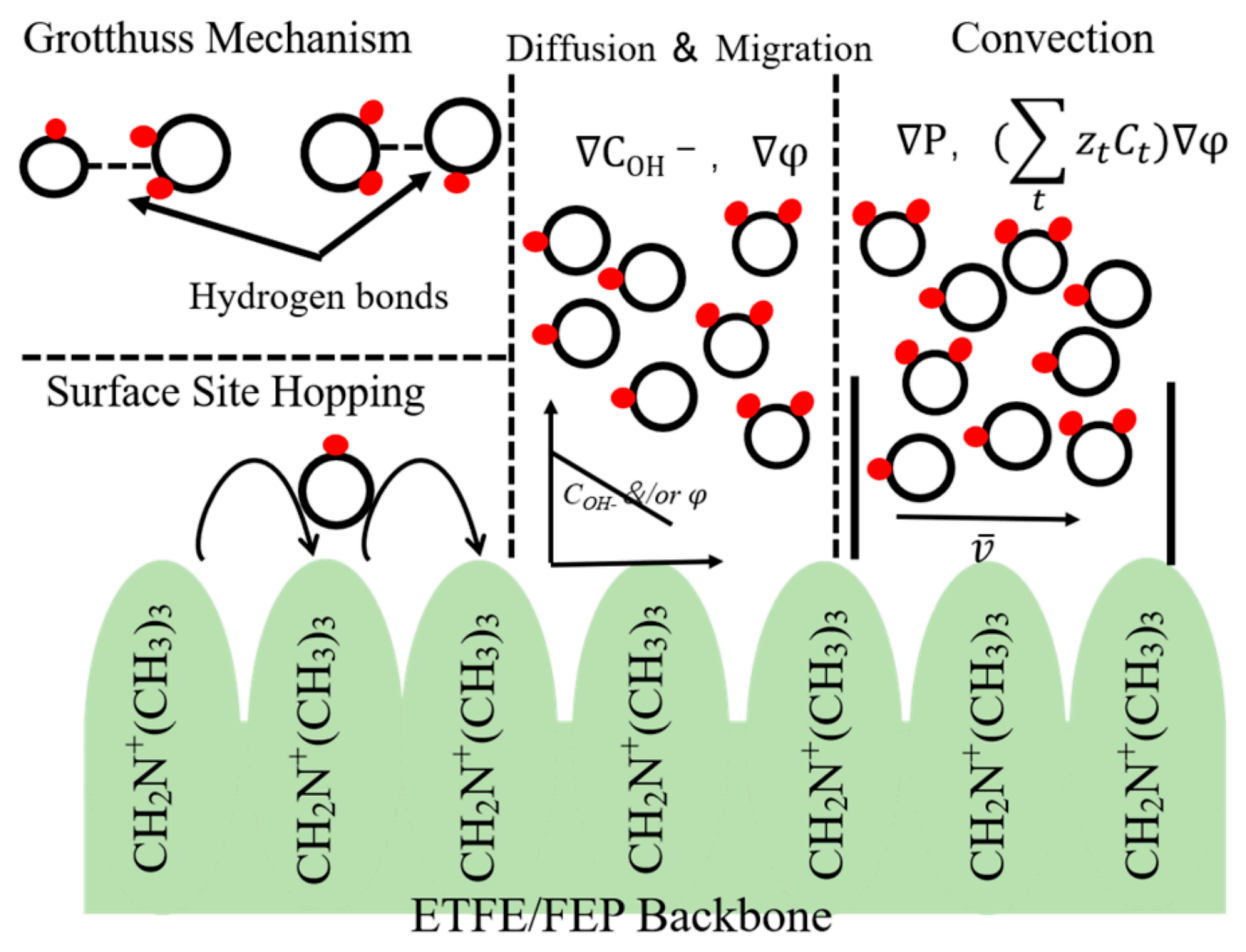
Illustration of OH^−^ transport mechanisms [10].

AEM is one of the main core components of AEMWE electrolytic cells. In the electrode reaction process, in addition to separating the cathode and anode, blocking electron shuttle, and gas diffusion, AEM mainly undertakes the task of transferring OH^–^ anions and water molecules. The transport mechanism of OH^–^ in AEM is generally believed to be similar to the proton conduction mechanism in proton exchange membranes (PEMs), mainly including the Grothuss mechanism, diffusion and migration mechanism, and convection mechanism (Figure 5 and Figure 6) [10]. The Grotes mechanism involves the formation of hydrated OH^–^ ion clusters through hydrogen bonding between OH^−^ and water molecules in a solution. When in close proximity to surrounding water molecules, hydrogen bonding breaks and rearranges, and then continues to form hydrated OH^–^ ion clusters with other water molecules. As hydrogen bonds continue to form and break, OH^–^ completes its transport in the electrolyte. The diffusion and migration mechanism is the movement of OH^–^ in aqueous solutions driven by concentration/potential gradients. The convection mechanism is due to the binding of OH^–^ ions with water molecules during transport, causing directional movement of water molecules and creating a water pressure gradient, resulting in convection phenomena. Under actual operating conditions, the OH^–^ transport behavior is the result of multiple transport mechanisms working together. As for which transport mechanism dominates, this is not only highly related to the molecular structure within the AEM ion conduction channel, but also largely depends on the amount of water in the membrane or the hydration number (*λ*) of the ion groups [11,35,36,37,38,39]. Meanwhile, it is also influenced by experimental conditions such as temperature, relative humidity, and pressure.

**Scheme 1:**
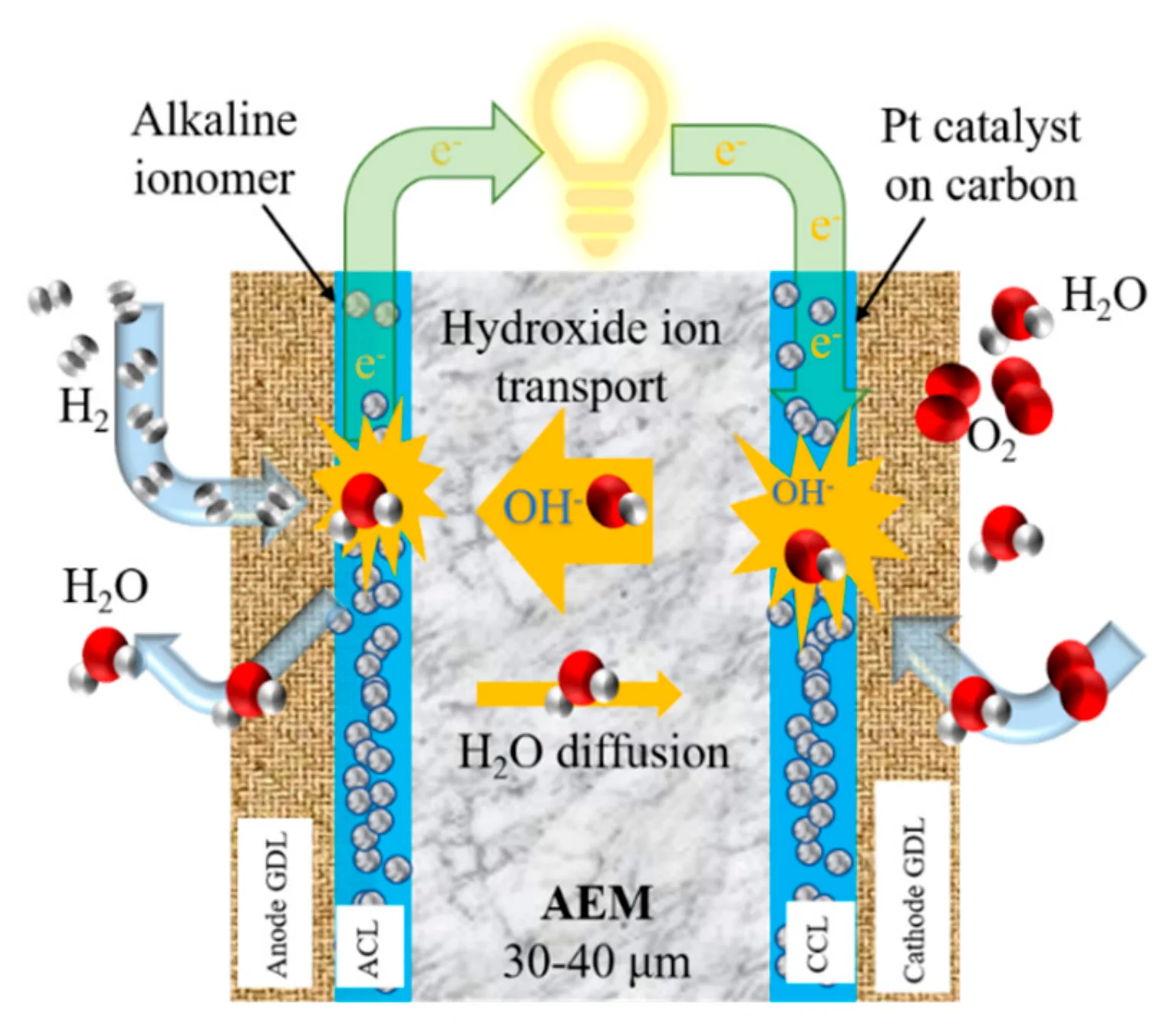
Schematic diagram of the operating principle of an alkaline water electrolysis cell.

A Scheme 1 illustrating the working principle of an alkaline water electrolysis cell, which produces green hydrogen through water electrolysis. Core Structure: At the center is a 30–40 μm-thick alkaline exchange membrane (AEM), which conducts hydroxide ions (OH^−^) while isolating the gases on either side; the left side is the anode and the right side is the cathode, both of which are coated with a carbon-supported platinum catalyst to accelerate the electrolysis reaction; The porous gas diffusion layers (GDLs) on both sides facilitate water diffusion and the release of hydrogen and oxygen. Workflow: Upon energization, water molecules lose electrons at the anode, producing oxygen, hydroxide ions, and protons. The hydroxide ions migrate toward the cathode through an alkaline exchange membrane. Upon reaching the cathode, the hydroxide ions combine with electrons to form hydrogen gas. The hydrogen gas generated by this reaction is discharged from the cathode, while oxygen is discharged from the anode, thereby achieving the separation of hydrogen and oxygen. Technical Features: Utilizing an alkaline environment reduces the corrosion resistance requirements for the catalyst, keeping costs relatively manageable. This is currently one of the key technical routes for green hydrogen production.

**Figure 7:**
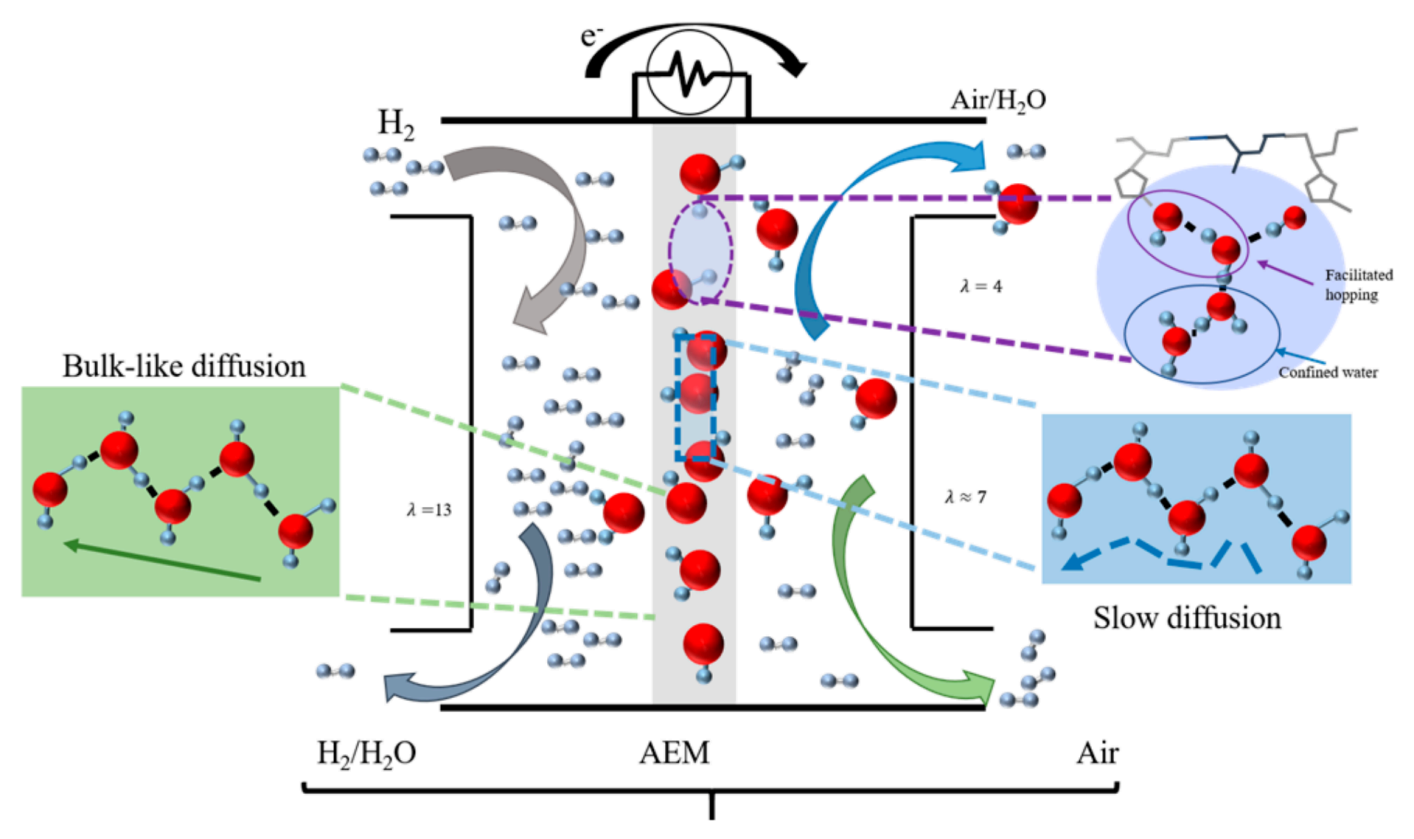
Schematic illustration of the main modes of OH^−^/H_2_O transport in different hydration regions of FAD-55 membrane [12].

In order to further clarify the transport mechanism of OH^–^ in AEMs, researchers have employed various technical methods to explore the kinetic process of OH^–^ in AEMs. For example, Chen et al. [64] constructed a multi-scale reaction molecular dynamics model using polyvinyl benzyl trimethylammonium (PVBTMA) as the research object. From the perspective of theoretical chemistry, the solvation structure and ion transport kinetics of OH^–^ ions in PVBTMA membranes were studied. The research results indicate that in the fully hydrated state of PVBTMA, OH^–^ is mainly transported from the overlapping regions between PVBTMA side chains, indicating that the transport mechanism plays a dominant role. Furthermore, it was confirmed that the activation energy of OH^–^ diffusion in AEM is greater than that of proton diffusion in PEM, and increasing the temperature is more conducive to the transport of OH^–^ in AEM. Ahn et al. [63] investigated the OH^–^ transport mechanism in a non-block polymer poly (p-phenylene ether) AEM using activated and non-activated polarized molecular dynamics simulations. Research has shown that OH^–^ mainly shuttles along sub-nanometer-wide water channels, and its mobility is significantly affected by “bottlenecks (narrow nodes)”. When OH^–^ is transported through these narrow nodes, if it is a transport mechanism, partial dehydration of OH^–^ is required, which requires higher activation energy and is thermodynamically disadvantageous. If it is the Grotes jump mechanism, OH^–^ moves from one side of the bottleneck to the other, the loss of hydration structure can be ignored, and the activation energy is very small. The balance between the size distribution of the rich water area and the bottleneck is a key factor determining the dominant role of the transport mechanism or Grotes mechanism. Foglia et al. [13] used quasi-elastic neutron scattering technology to investigate the water, polymer relaxation, and OH^–^ diffusion kinetics in commercially available Fumatech FAD-55 AEMs, as well as their synergistic effects (Figure 7). Research has shown that water is unevenly distributed between the cathode and anode of an electrolytic cell, and the transport mechanism of OH^–^ is largely determined by λ. Under the condition of complete hydration (λ = 13), the OH^–^ shuttle process is mainly dominated by the transport mechanism, with the Grotes mechanism accounting for about 15% of the entire process, and the self-diffusion coefficient of moving particles in the solution is equivalent to the total diffusion coefficient of the membrane. When the degree of hydration decreases to λ = 7, due to the interaction between water and the polymer matrix, the self-diffusion coefficient is about 50% slower than when λ = 13. When λ ≤ 4 (at high current densities, λ can decrease to 1), the transport behavior of OH^–^ mainly follows the Grotes mechanism. These research results not only contribute to a deeper understanding of the transport behavior of OH^–^ in AEMs, but also provide guidance for the structural design of high-performance AEMs.

### 3.2. Performance Requirements for Anion Exchange Membranes

The electrolytic performance of AEMWE largely depends on the performance of AEMs, therefore high-performance AEMs should meet the following conditions [36,37,38,39,40,41]: (1) high OH^−^ ion conductivity helps to reduce the ohmic resistance of the electrolytic cell and improve battery efficiency; (2) good alkali resistance stability ensures that the membrane material can operate for a long time without degradation under high-temperature and strong alkali conditions; (3) excellent dimensional stability, thermal stability, and mechanical strength enable the membrane to withstand higher pressure differentials; (4) good gas barrier to avoid cross permeation of H_2_ and O_2_, which can cause battery performance degradation and safety issues; (5) appropriate water absorption and swelling degree ensure the formation of continuous hydroxide ion transfer channels within the membrane, avoiding excessive swelling and loss of dimensional stability caused by excessive water content; (6) the cost of membrane raw materials is low, easy to process, and easy to prepare on a large scale. Among all the performance requirements mentioned above, stability is a prerequisite for AEM applications, and sufficiently high ion conductivity is the key to achieving high electrolysis performance [35,37,38,39,40,41].

The main parameters for evaluating AEM performance include ion exchange capacity (IEC), water absorption, swelling ratio λ, ionic conductivity, alkali stability, H_2_/O_2_ gas permeability, and single-cell testing. Introducing more cationic groups into the AEM main chain is beneficial for improving IEC and ionic conductivity, but it can lead to an increase in water absorption and swelling ratio, affecting the size and mechanical stability of the membrane. Highly stable cationic groups and polymer main chains can enhance the alkali stability of the membrane.

## 4. Research Progress on Anion Exchange Membranes

**Figure 8:**
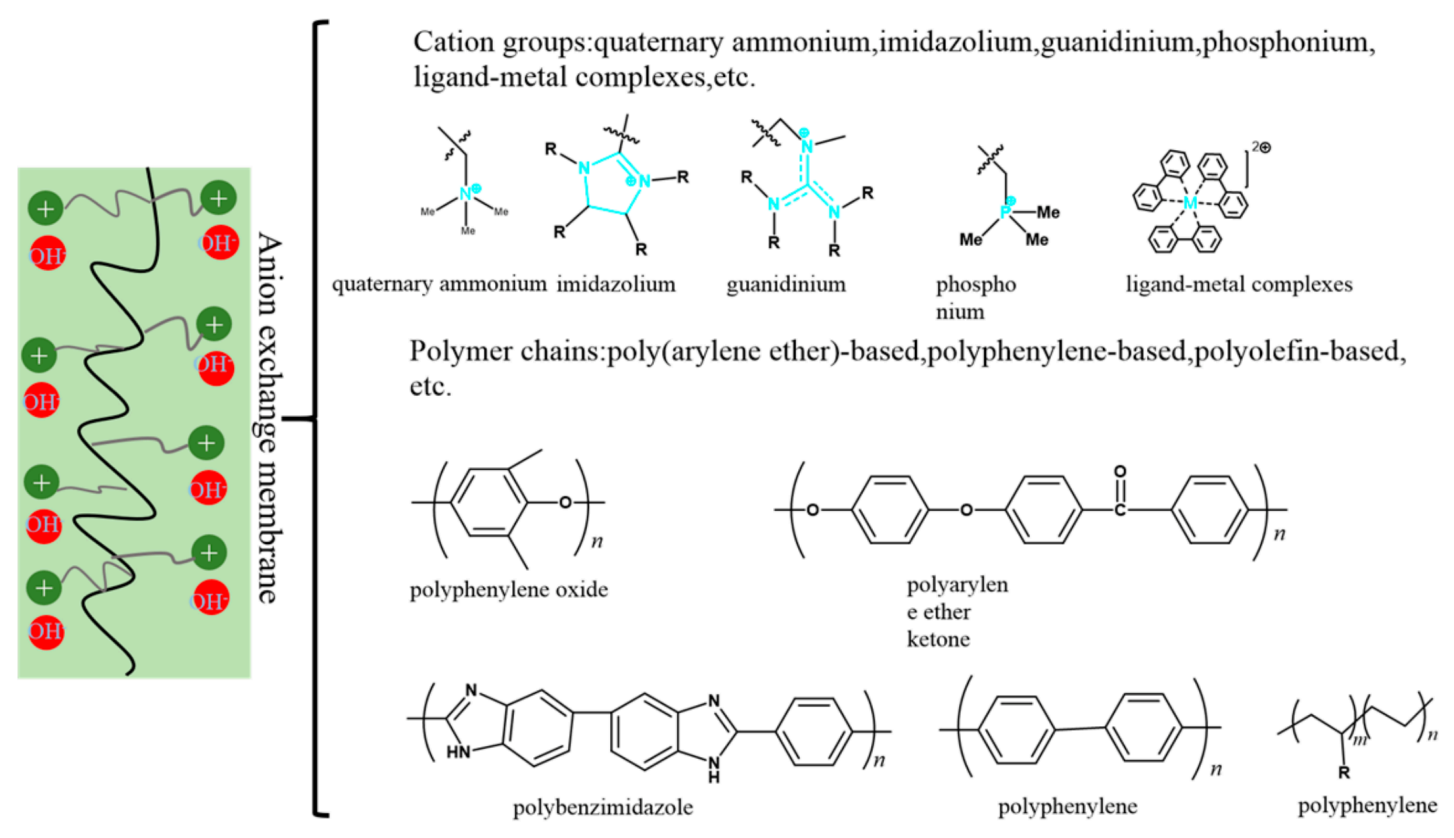
Structure of AEM, including the cation groups and polymer chains.

An AEM is usually composed of two parts: cationic groups and polymer main chains. Cationic groups are functional groups connected to the polymer main chain, responsible for the conduction of OH^−^ and determining the ion conductivity, water absorption, and swelling rate of the AEM. The polymer backbone, as the main supporting material of the membrane, determines the dimensional stability and mechanical strength of the membrane [13]. Common cationic groups include quaternary ammonium cations, imidazole cations, quaternary guanidine cations, quaternary cations, and metal cations; The main chains of polymers include polyarylethers (such as polyphenylene ether, polysulfone, polyetheretherketone, etc.), and polyaryls without ether oxygen bonds (such as polyphenylene, polybenzimidazole, arylimidazoles, polyfluorenes, polyolefins (such as polyethylene and cyclic olefin polymers, etc.) (Figure 8) [14]. In recent years, researchers have done a lot of research work to develop high-performance AEMs. The main research progress of AEMs is explained below from the design of cationic groups and polymer backbones.

### 4.1. Cationic Group Structure Design

#### 4.1.1. Quaternary Ammonium Cation

**Figure 9:**
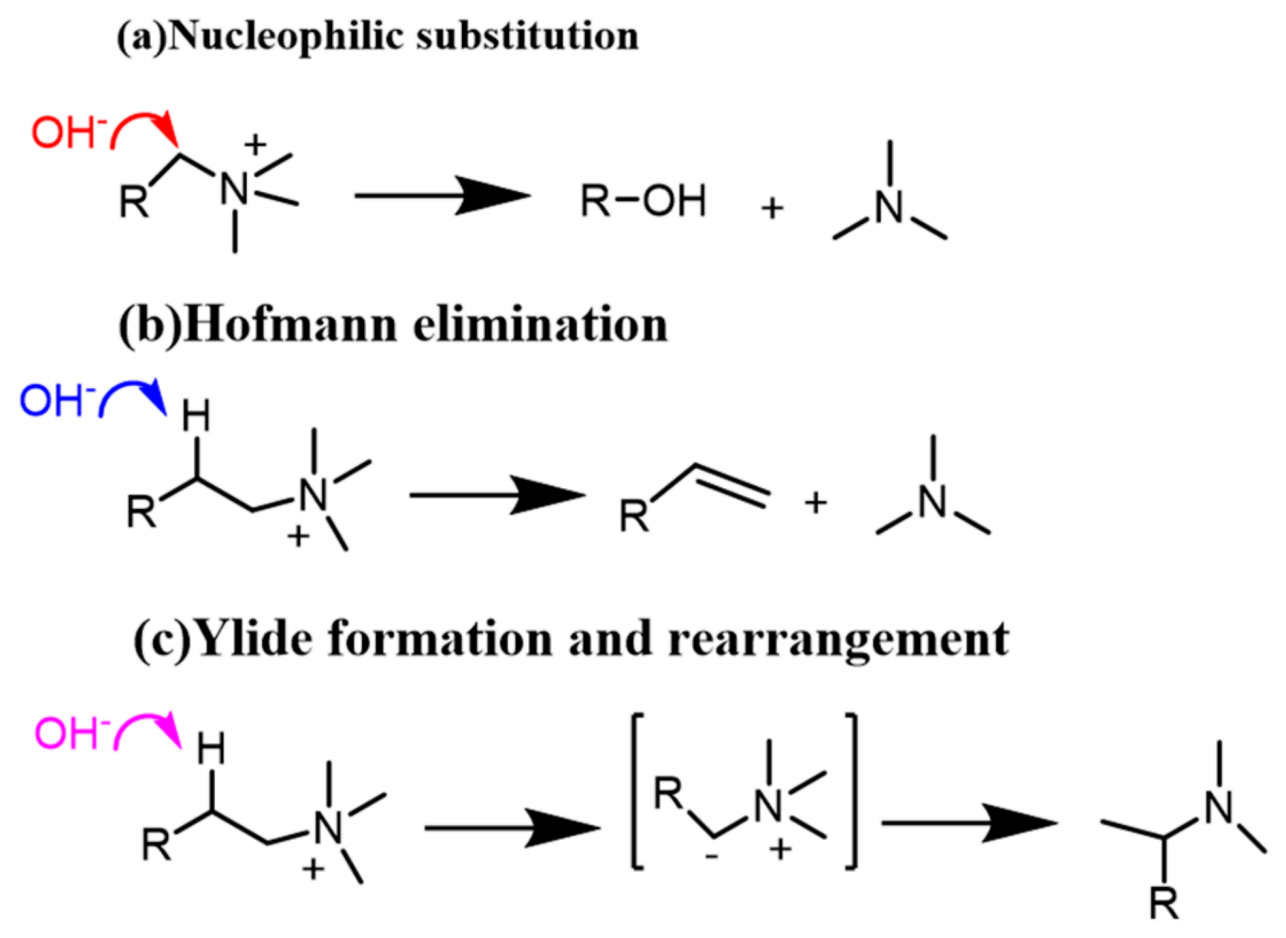
The degradation mechanism of quaternary ammonium cation groups [14].

Quaternary ammonium cations have the advantages of simple preparation, low cost, and high ionic conductivity, making them the most widely used cations in AEMs. However, quaternary ammonium cations are prone to degradation in high-temperature and high alkaline environments, and the main degradation pathways are shown in Figure 9 [14]: (1) *α*-C is easily attacked by OH^−^, undergoes nucleophilic substitution reactions, and degrades into tertiary amines and alcohol by-products; (2) *β*-H is attacked by OH^−^; Hofmann eliminates the reaction and degrades it into olefins and tertiary amine by-products; (3) after being attacked by OH^−^, *α*-H forms an N-ylide intermediate, which then undergoes rearrangement (Sommelet Hauser and/or Stevens rearrangement) to produce more stable tertiary amines. In order to improve the alkali stability of quaternary ammonium cations, researchers usually introduce substituents with greater steric hindrance on the quaternary ammonium cations, which can effectively reduce the attack of OH^−^ and improve the alkali stability of AEM. For example, Li et al. [53] introduced alkyl chains of different lengths onto quaternary ammonium ions using the Menshutkin reaction, which increased the steric hindrance effect around the quaternary ammonium ions, effectively weakened the attack of OH^−^, and inhibited the Hofmann elimination reaction. After soaking at 80 °C and in 1 mol/L NaOH for 2000 h, the series of membranes still maintained acceptable mechanical properties. Dresp [69] further investigated the alkali stability of quaternary ammonium cations containing different types of substituents at 160 °C and 10 mol/L NaOH solution. The results indicate that the stability of quaternary ammonium ions modified with electron-donating groups is higher than that of quaternary ammonium ions modified with electron-withdrawing groups. Quaternary ammonium ions with benzyl groups are more susceptible to attack by nucleophiles and have poorer stability. Meanwhile, heterocyclic substituted quaternary ammonium cationic groups such as N, N-dimethylpiperidine (hexagonal ring) and N, N-dimethylpyrrolidine (pentagonal ring) exhibit higher stability in alkaline solutions. Zhang et al. [73] also obtained similar results by combining experiments and density functional theory (DFT) calculations, that is, the contribution of substituents to the stability of quaternary ammonium cations from high to low is: electron-donating groups, unsubstituted groups, and electron-withdrawing groups. In addition, if *β*-C does not contain hydrogen atoms, it can effectively avoid the occurrence of Hofmann elimination reaction, which is beneficial for the stability of quaternary ammonium cation groups. For example, Wu et al. [39] prepared a bis quaternary ammonium cationic group AEM without *β*-H through Cu (I)-catalyzed azide alkynyl click chemistry reaction. After being immersed in a 2 mol/L NaOH solution (80 °C) for 480 h, the membrane exhibited excellent alkaline stability and high conductivity retention rate (>92%).

#### 4.1.2. Imidazole Type Cations

The imidazole cationic group has attracted widespread attention from researchers due to its simple synthesis method and strong structural design ability [19]. Under alkaline conditions, imidazole cations are usually degraded through four pathways, which are closely related to the properties of the substituents (Figure 9) [14]: (1) when the *C*_2_ position is H, it is easy to degrade through ring opening reactions; (2) when H is present at positions *C*_4_ and *C*_5_, degradation occurs through heterocyclic deprotonation reaction; (3) when methyl or benzyl groups are attached to N_1_ and N_3_, degradation occurs through nucleophilic substitution reaction mechanism; (4) when N_1_ is benzyl, it is degraded through deprotonation reaction with substituents. Based on the degradation mechanism of imidazole cations, researchers can effectively prevent the reaction between reactive ions in solution and imidazole cations by adding suitable substituents or modifying functional groups at different sites of imidazole cations, thereby enhancing the alkali stability of imidazole cations. Research in [21] studied the effect of introducing methyl, isopropyl, and phenyl groups at the *C*_2_ position on the stability of imidazole cations employing NMR. The results indicate that imidazole cations with substituents at the *C*_2_ position exhibit better chemical stability than those without substituents. This may be due to the steric hindrance effect and *γ*-π hyper-conjugation effect of the substituted imidazole cation, which inhibits the attack of OH^−^. The results of DFT simulation calculations indicate that imidazole cations with the lowest unoccupied molecular orbital energy level have high chemical stability. Wang et al. [4] studied the effect of introducing methyl groups at *C*_4_ and *C*_5_ positions on imidazole cations. Due to the effects of electron supply and steric hindrance, the ionic conductivity and stability of imidazole cations have been improved. In addition to *C*_4_ and *C*_5_ substitution of imidazole rings, the alkali stability of imidazole cations may also be affected by N_3_ substitution. Gao et al. [17] used DFT simulations to investigate the effects of different alkyl groups (such as methyl, ethyl, and isopropyl) introduced at the N_3_ position on imidazole cations to investigate the effects of different alkyl groups on imidazole cations. Compared with traditional quaternary ammonium cations, imidazole cations substituted at the N_3_ position exhibit higher chemical stability, with isopropyl-substituted imidazole cations showing the best stability and displaying the highest lowest unoccupied molecular orbital energy level. This is related to the increase in electron density of imidazole cations caused by the formation of hyper-conjugation and electron-donating effect after alkyl substitution, as well as the increase in steric hindrance of imidazole cations. Ma et al. [58] reported several imidazole cationic compounds with higher steric hindrance and conducted half-life tests in a 3 mol/L NaOD/D_2_O/CD_3_OD solution at 80 °C. The results showed that the half-lives of four highly hindered imidazole cations were all greater than 10,000 h, and their stability was much higher than that of common quaternary ammonium cations and small-hindered imidazole cations. Although introducing substituents on imidazole cations can improve their stability in high-temperature alkaline solutions, the preparation process of this modification is complex and can affect the film-forming ability and mechanical strength of AEMs, which has certain limitations.

#### 4.1.3. Quaternary Guanidine Cation

**Figure 10:**
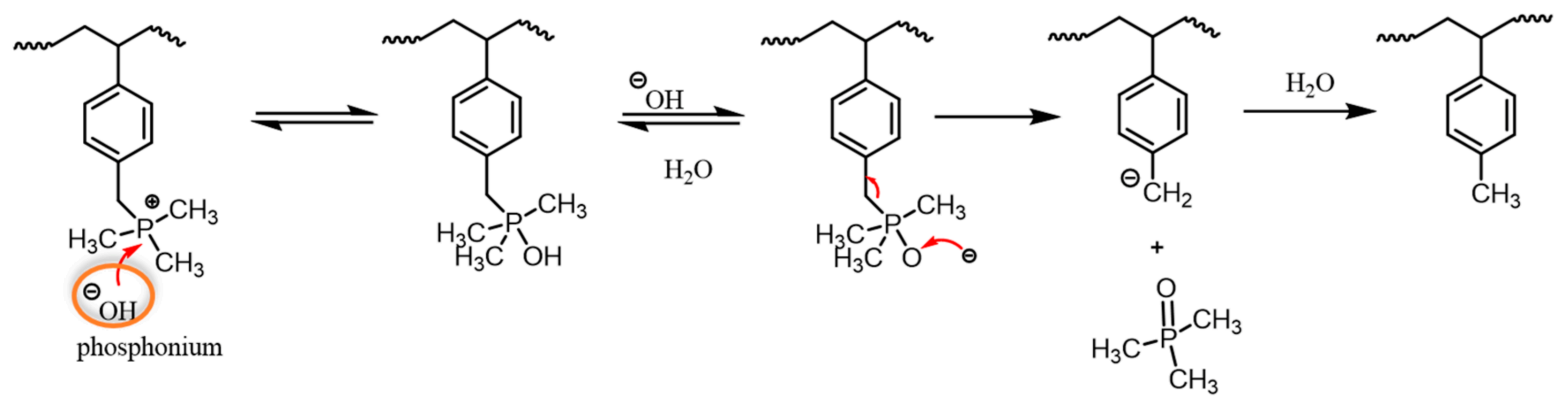
Degradation routes of quaternary phosphonium cation group [26].

Quaternary guanidine cation was first applied in AEMs in 2010 [25]. Research has found that under alkaline conditions, quaternary guanidine cations are mainly degraded through nucleophilic addition elimination (Figure 10), where the central *C* atom is attacked by OH^−^ and undergoes an addition reaction to form an intermediate, followed by protonation of the N atom and elimination reaction to form degradation products urea and amine [26]. Therefore, introducing different substituents and increasing steric hindrance effect can enhance the stability of quaternary guanidine cations. Xue et al. [15] used ^1^H NMR to study the alkaline stability of a series of guanidine cations containing different N-substituted groups. They found that the contribution order of substituents to the stability of guanidine cations was: isopropyl > ethyl > benzyl > phenyl, ethyl > methyl > cyclobutyl. A quaternary guanidine cationic polysulfone AEM with this functionalization was prepared through the Suzuki–Miyaura reaction. After soaking the membrane in a 1 mol/L NaOH solution at 60 °C for 30 days, no degradation was observed, indicating that the quaternary guanidine cation AEM with large steric hindrance has excellent alkali stability. Subsequently, the author used brominated poly (2,6-dimethyl) p-phenylene oxide and biguanide crosslinking agent to prepare AEM-containing biguanide cation through Menshutkin reaction. The test results indicate that the AEM crosslinked with biguanide also has good tolerance to alkaline solutions and can effectively control the swelling of the membrane [25].

#### 4.1.4. Quaternary Cations

As a member of the same group of elements as N, the P element has similar chemical properties to the N element, so the quaternary cation AEM has long been of interest to researchers [26]. However, under alkaline conditions, quaternary cations are prone to undergoing the phosphorite reaction, leading to the decay of the AEM (Figure 10) [26]. In order to solve the problem of poor stability, researchers have conducted modification studies on quaternary cations using electron-donating groups or large steric hindrance groups. For example, Grew et al. [10] prepared AEMs with large steric hindrance of quaternary cations. After soaking the AEM in a 15 mol/L KOH solution at 22 °C for 20 weeks, there was no significant decrease in the ion conductivity of the membrane. After soaking in a 1 mol/L KOH solution at 80 °C for 3 days, the ion conductivity of AEM decreased from 22 mS/cm to 18 mS/cm, but no decrease was observed in the ion conductivity of the membrane after 19 days. Zhang et al. [73] prepared an AEM by attaching 2,4,6-trimethoxyphenyl to quaternary cations. The membrane was immersed in a 1 mol/L KOH solution at room temperature for 30 days, with ion conductivity of 27 mS/cm. After soaking in a 1 mol/L KOH/(CD_3_OD + D_2_O) solution at 80 °C for 5000 h, ion conductivity of the membrane decreased by about 20%, demonstrating good alkali stability.

#### 4.1.5. Metal Cations

Due to the absence of nucleophilic substitution of organic cation groups, Hofmann elimination reaction, and Yehead reaction degradation pathways in metal cations, researchers have studied AEMs based on metal-based cations [29]. The metal cations formed by transition metals and complexes, such as ruthenium triphenylpyridine [30], nickel triphenylpyridine [31], and cobalt ferrocene [32], exhibit excellent alkaline stability and ionic conductivity. Liu et al. [18] reported a ferrocene functionalized AEM, which showed no significant decrease in ion conductivity after soaking in a 1 mol/L NaOH solution at 95 °C for 4320 h. However, considering the redox stability of transition metal cations, metal cations formed through the coordination of main group metals and ligands are applied in AEMs. Chen et al. [11] prepared AEM-containing sodium metal cations based on Na^+^ and ligand benzo-15-crown-5. After soaking the membrane in 4 mol/L NaOH at 60 °C for 10 days, the decay rate of ion conductivity was only 2.5%, demonstrating good alkali stability. Zhao et al. [51] studied an AEM based on ligand [2.2.2] ether complexation with metal Ba^2+^. The test results showed that the AEM did not show significant degradation after soaking in a 15 mol/L KOH strong alkaline solution at 60 °C for 1500 h, demonstrating excellent alkaline stability. However, due to the complex preparation process, difficult grafting, and high cost of metal cations, their application in AEMs is limited.

### 4.2. Polymer Main Chain Design

#### 4.2.1. Polyarylether-Based Main Chain

The main chain of polyarylene ether polymers has strong hydrophobicity, which can reduce the probability of degradation when in contact with OH^−^ and exhibit strong alkali stability. This type of polymer backbone also has the advantages of excellent thermal stability, good mechanical properties, and low preparation cost, and has received widespread attention from AEM researchers. At present, the strategies for modifying the main chain of poly (arylene ether) include cationic functionalization, crosslinking, copolymerization, etc.

Due to the presence of benzene rings and ether bonds on the main chains of polyphenylene ether, polyether sulfone, and polyether ether ketone, it is easy to prepare AEMs containing different cations by introducing halide sites through alkylating reagents. Zhu et al. [25] used the bromination reagent N-bromosuccinimide to undergo a free radical reaction with polyphenylene ether, introducing bromination sites at the benzyl group. Then, a series of quaternary ammonium-functionalized polyphenylene ether AEMs were prepared through Menshutkin reaction with trimethylamine or imidazole. Liu et al. [18] prepared a quaternary ammonium ion AEM based on polyether sulfone by reacting the phenyl ring of polyether sulfone with triethylamine and introducing a quaternary ammonium cation. They found that the degree of chloromethylation was closely related to the physical and chemical properties of the membrane.

The use of crosslinking agents to crosslink the main chain of polyarylether polymers through side chains can effectively improve the aqueous ion absorption, ion conductivity, dimensional stability, and mechanical strength of AEMs. However, excessive crosslinking can lead to a decrease in membrane flexibility. Research in [37] introduced N, N-dimethylallylamine- and N-allylmethylamine-containing unsaturated double bonds as side chains onto the main chain of polysulfone, and then prepared polysulfone-based AEM through heating-induced crosslinking. The research results confirm that the membrane not only improves mechanical/dimensional stability, but also enhances ion conductivity. Sung et al. [38] reported a series of hydrophilic crosslinking agents such as dimethylaminoethyl ether.

Block copolymerization is a new method that can effectively improve ion conductivity. By connecting polyphenylene ether, polyether sulfone, and polyether ether ketone with other monomers through chemical bonds to form block copolymers, the ion conductivity and dimensional stability of the membrane can be effectively regulated. Research in [45] showed the synthesizing of a copolymer AEM using polyphenylene ether and hydrogenated styrene butadiene and investigated the effect of triazole on the membrane. The results of X-ray diffraction and atomic force microscopy show that compared with the copolymer film without triazoles, the copolymer AEM film prepared with triazoles has better OH^−^ conductivity, mechanical stability, and alkali resistance due to rapid phase separation, and exhibits higher current density at 1.8 V. These changes may be related to the strong hydrogen bonding between triazoles and water/OH^−^. Within the block copolymer, due to the presence of hydrophilic/hydrophobic microphase separation morphology, ion transport channels can be formed, which is beneficial for improving ion conductivity.

**Figure 11:**
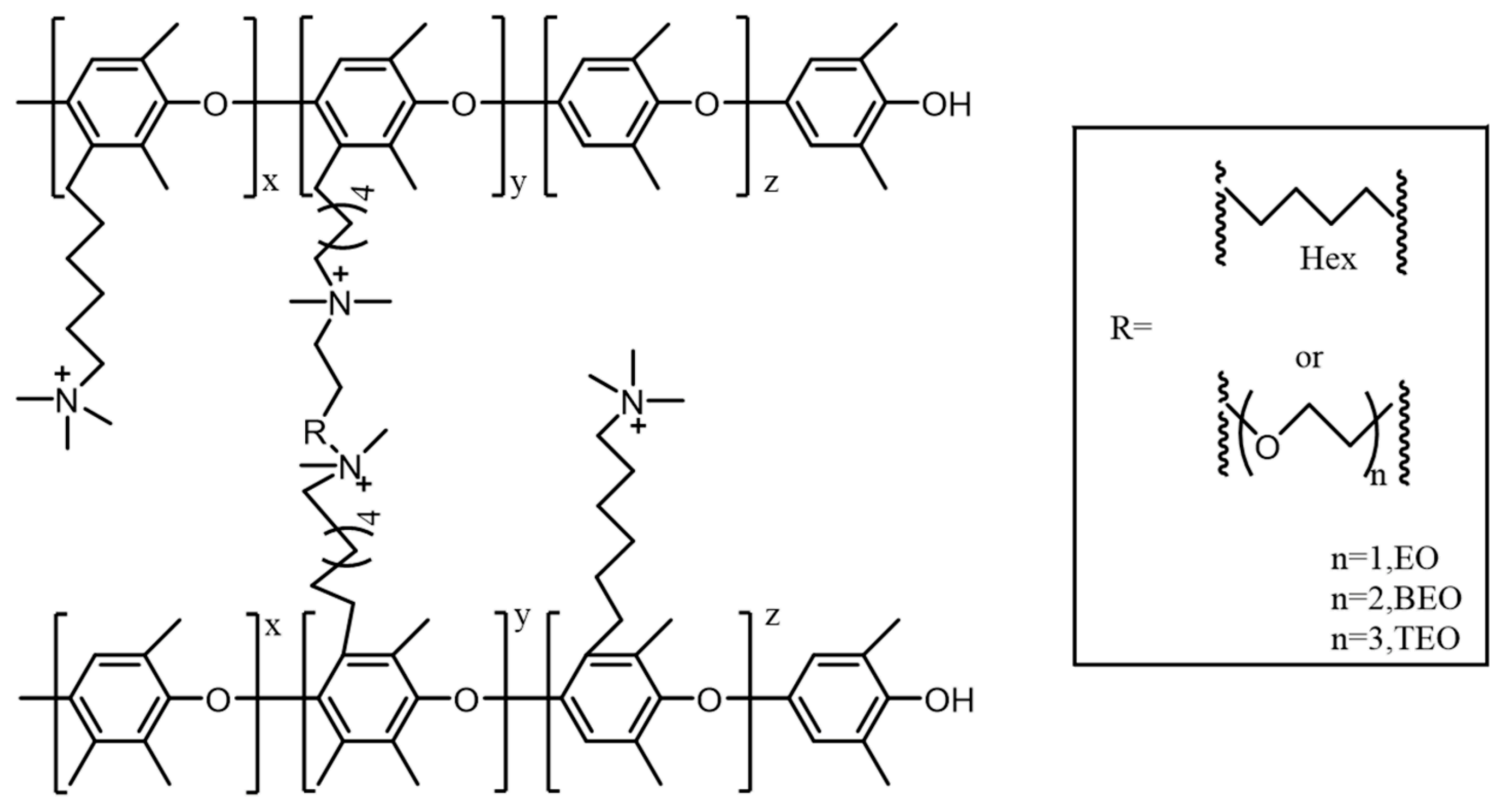
Structure of the AEM crosslinked with polyphenylene oxide [37].

The preparation of quaternized AEM based on polyphenylene ether crosslinking is shown in Figure 11. The test results showed that the series of membranes achieved the highest ion conductivity of 131.96 mS/cm in 80 °C water, and the peak power density of the single cell was 444 mW/cm^2^ (677 mA)/cm^2^@0.6 V) However, while hydrophilic crosslinking agents provide high water absorption capacity to improve the conductivity of AEMs, they also lead to the formation of polymer film crystals, reducing the alkali stability of AEMs. In order to further investigate the effect of crosslinking agent content on membrane properties, research in [38] used ethylene glycol (PEG) as a crosslinking agent to prepare quaternized polyetherketone-based (QPPEEK) AEMs with different crosslinking agent contents. The research results show that the mechanical properties and ion conductivity of the membrane can be adjusted by the mass fraction of QPPEEK and PEG components. When the mass ratio of QPPEEK to PEG is 80:20, the membrane exhibits the best performance with ion conductivity of 10^2^ mS/cm (80 °C) and a tensile strength of 13.7 MPa. After being treated in a 1 mol/L KOH solution at 80 °C for 400 h, the AEM still maintained 85% of its initial conductivity.

Although polyarylether-based main chains exhibit excellent performance in terms of preparation cost, alkali/thermal stability, and mechanical strength, and some of their properties can be further improved through modification, the presence of aromatic ether bonds in the polymer skeleton makes it easy for the main chain to degrade under alkaline conditions when attacked by OH^−^, resulting in a decrease in mechanical strength [42]. In addition, if the main chain of polyaryletheric ether contains electron-withdrawing cationic groups, it can accelerate the degradation of the main chain [43]. Therefore, researchers have begun to focus on the development of ether oxygen-bond-free polymer main chains.

#### 4.2.2. Non-Ether Oxygen-Bonded Aromatic Main Chain

**Figure 12:**
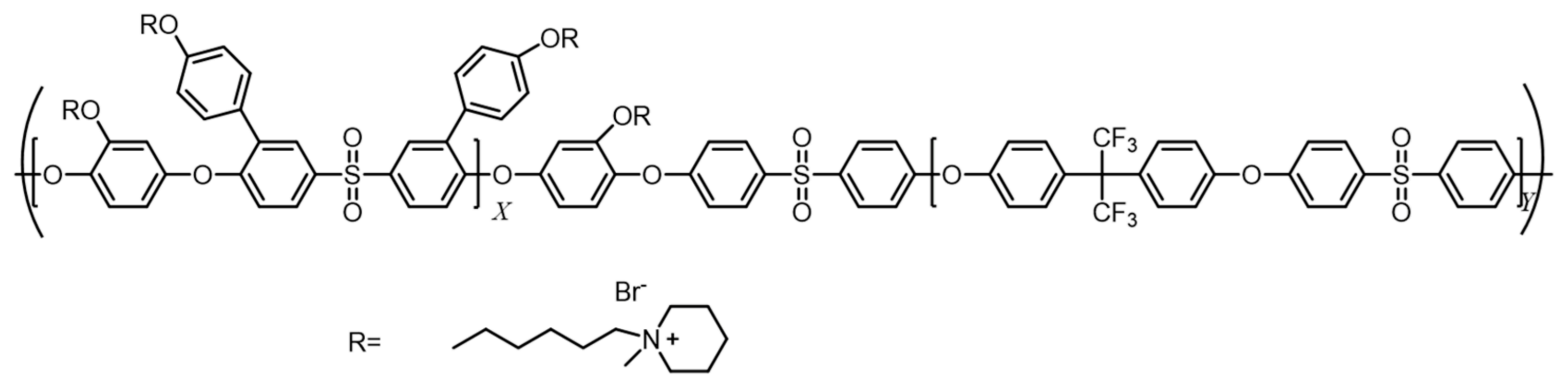
The structure of multi-block poly (ether sulfone) AEM [40].

The main chain of aromatic polymers without ether oxygen bonds has the advantages of good alkali stability, high structural rigidity, excellent dimensional stability, and high thermal stability. Polymers containing non-ether oxygen-bonded aromatic main chains mainly include polyphenylene, polybenzimidazole, polyarylimidazole, and polyfluoranthene. Park et al. [61] prepared a quaternized polyphenyl AEM via the Diels Alder reaction and investigated the effect of different side chain lengths on alkali stability. The research results indicate that a quaternary ammonium polyphenyl AEM, which is separated from the main chain by six methylene groups, exhibits better alkali stability in a 4 mol/L KOH solution at 80 °C. Shen et al. [41] prepared a multi-block polyether sulfone copolymer-based AEM containing flexible side chain piperidine cationic groups using the end-group crosslinking method (Figure 12). The research results showed that the membrane exhibited an ionic conductivity of 105.4 mS/cm at 80 °C, while also demonstrating good thermal stability and alkali resistance.

**Figure 13:**
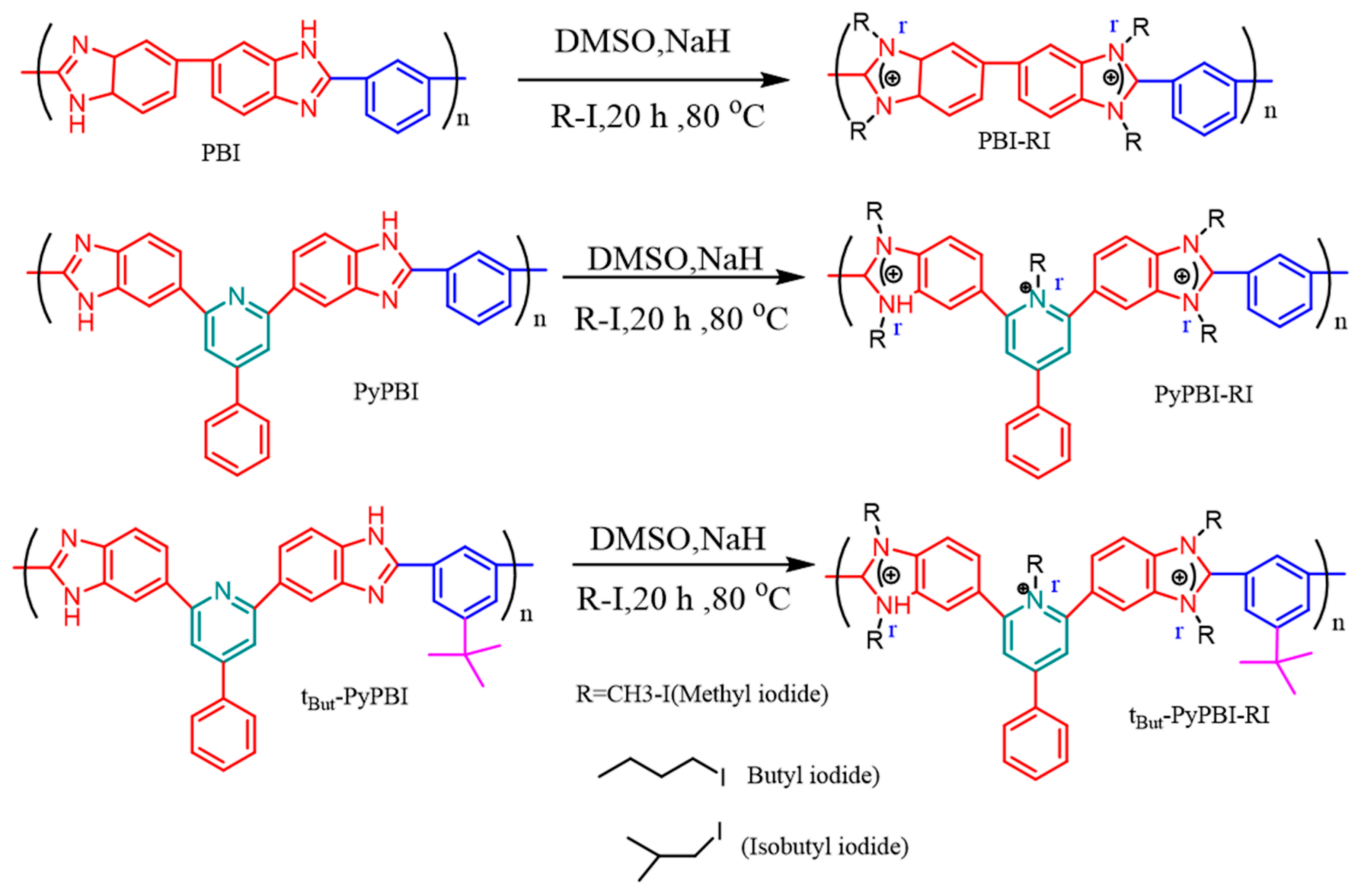
Synthesis of polybenzimidazole electrochemical membrane (AEM) [47].

Sana et al. [45] converted three different types of polybenzimidazole polymers into their iodide forms through the reaction of imidazole ring and alkyl iodide reagents (such as methyl iodide, butyl iodide, and isobutyl iodide), and then immersed them in KOH solution to obtain a polybenzimidazole-based AEM (Figure 13). The effects of different alkyl side chains on the IEC, ionic conductivity, thermal, mechanical, and alkaline stability of the obtained polybenzimidazole-based AEM were investigated [44,49]. This indicates that an AEM with alkyl side chains has good alkaline stability and higher ion conductivity. Among them, the AEM membrane with butyl iodide as the alkyl side linking branch showed the highest ion conductivity of 128.6 mS/cm at a temperature of 80 °C. All polybenzimidazole-based AEMs containing isobutyl and butyl side chains exhibit excellent alkali stability in a 5 mol/L KOH aqueous solution at 60 °C. This is mainly due to the presence of alkyl side chains, which prevent OH^−^ from attacking the pyridine and imidazole ion groups.

**Figure 14:**
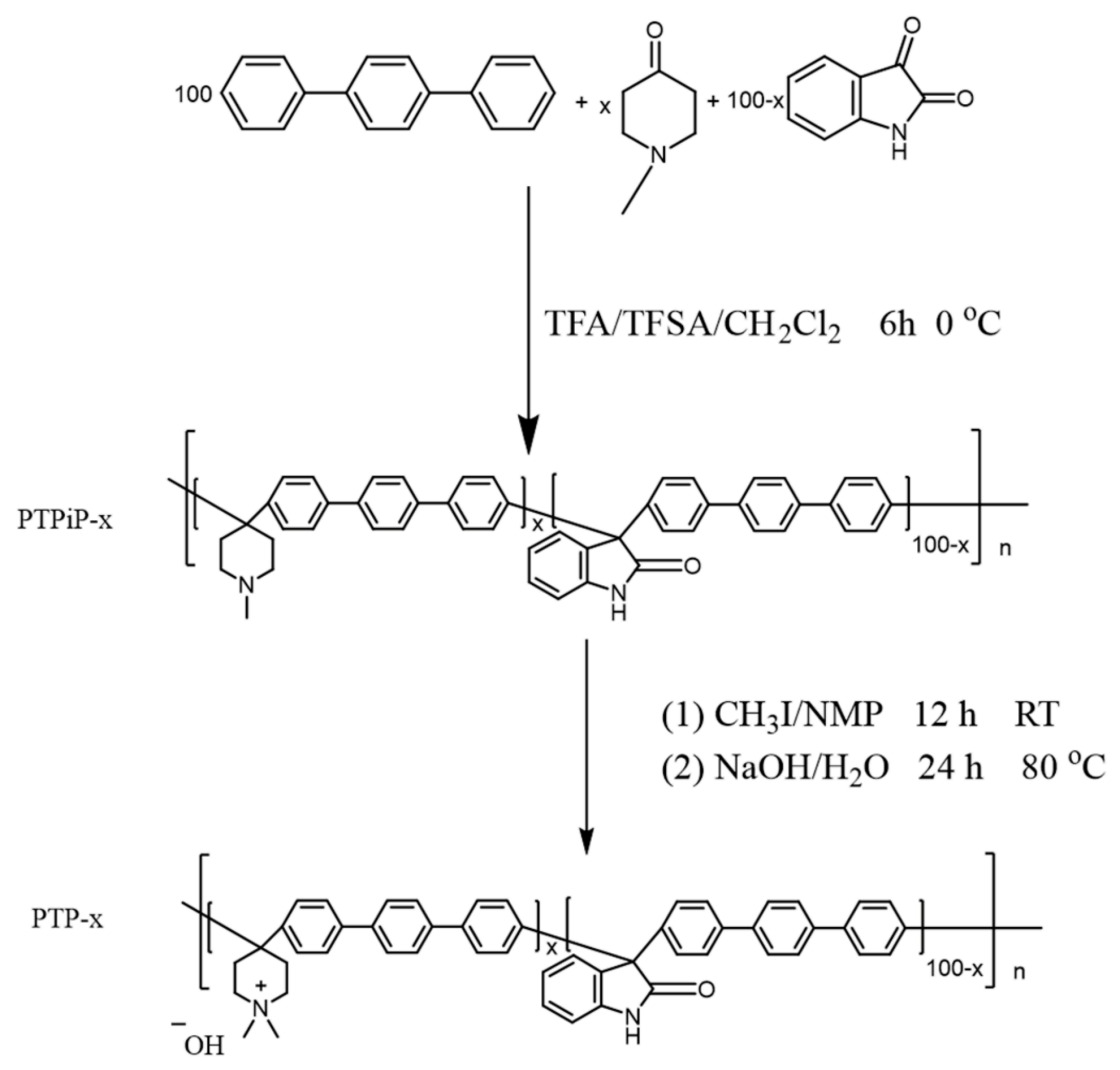
Synthesis of poly [(terphenyl piperidinium)-co-(oxindole terphenylylene)](PTP) AEM [48].

With the use of superacid-catalyzed condensation reactions for the preparation of ether-free aromatic AEMs, it has received widespread attention from researchers. This reaction is catalyzed by superacids, where the carbonyl group is protonated and then used as an electrophilic reagent to attack the aromatic ring, leading to alkylation and the formation of ether-free aromatic polymers. Chen et al. [64] synthesized a series of poly (fluorene co terphenyl *N*,*N*′-dimethylpiperidinium) PFTP-*x* copolymers using superacid-catalyzed condensation reaction at −3 °C with an AEM (where x is the molar ratio of fluorenylpiperidine segments in the copolymer). PFTP-*x* membrane snot only have a simple synthesis process, but also have excellent ion conductivity, mechanical/dimensional stability, dimensional stability, and excellent film-forming ability, among which PFTP-13 has the best comprehensive performance. The mechanical/dimensional stability of a PFTP-13 AEM is excellent, with a tensile strength of up to 84.6 MPa, a Young’s modulus of 1580 MPa, and a fracture elongation of only 25.6%. It is also easy to produce films with a thickness of 7–20 μm. After soaking in 1 mol/L NaOH at 80 °C for 2000 h, the PFTP-13 membrane still maintains transparency and good mechanical toughness, with a loss rate of only 3% of the ion conducting group piperidinium, and its ion conductivity is as high as 208 mS/cm. Further research results indicate that the fluorene chain segment can improve the water channel and microphase separation morphology of PFTP-*x*, which is beneficial for enhancing the size stability and conductivity of the membrane. Hu et al. [43] prepared a series of poly (arylpyridine) (PTP) materials as AEMs for electrolytic cells using triphenylene, *N*-methyl-4-piperidone, and indigo carmine as raw materials, catalyzed by superacid trifluoromethanesulfonic acid (TFSA) and quaternized with methyl iodide (Figure 14). The polymer skeleton of this PTP material exhibits excellent alkali stability and strong mechanical properties (tensile strength reaching 29.2–36.5 MPa). The highest ion conductivity of PTP-90 membrane material obtained at 80 °C was 128.9 mS/cm; After treatment in a 1 mol/L NaOH solution at 80 °C for 934 h, the ion conductivity retention rate was 60%. Using an electrolytic cell equipped with PTP-90 membrane for testing, it was confirmed that the current density of the battery at 55 °C and 75 °C reached 910 mA/cm^2^ and 1000 mA/cm^2^, respectively, at a voltage of 2.2 V. Further durability testing revealed significant chemical degradation of the PTP-90 membrane after continuous electrolysis at 400 mA/cm^2^ and 55 °C for 120 h. This is mainly related to the degradation of quaternary ammonium cationic groups through Hofmann elimination reaction and nucleophilic substitution reaction, as well as the possible acceleration of the degradation of cationic groups in an AEM by the working environment of the electrolytic cell. From this, it can be seen that further research is needed on the durability of AEM in electrolytic cells under actual operating conditions.

With the use of superacid-catalyzed condensation reactions in the preparation of ether-free aromatic AEMs, it has received widespread attention from researchers. This reaction is catalyzed by superacids, where the carbonyl group is protonated and then used as an electrophilic reagent to attack the aromatic ring, leading to alkylation and the formation of ether-free aromatic polymers.

## 5. Conclusions and Prospect

**Figure 15:**
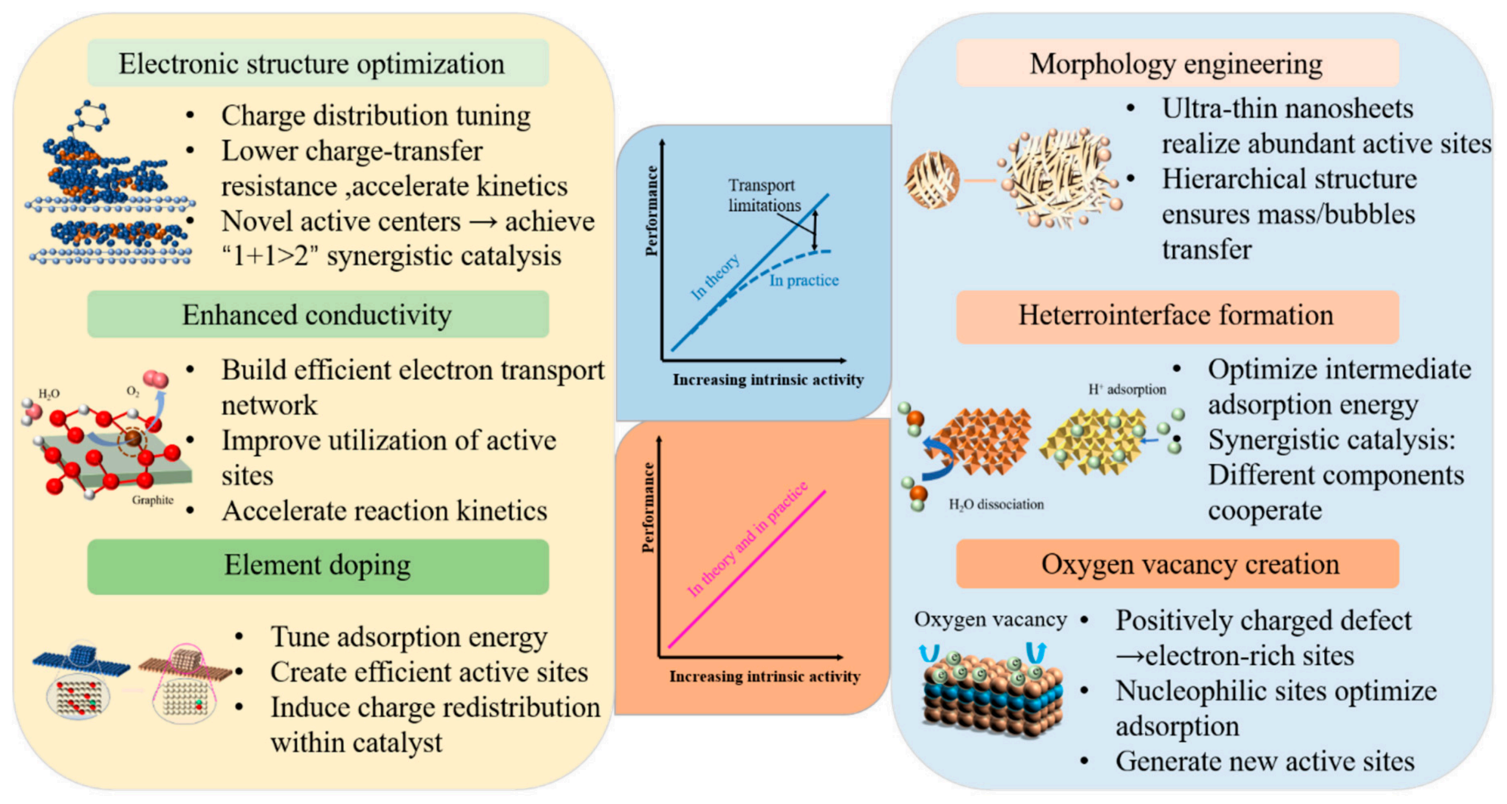
Illustration of strategies to increase catalytic performance.

AEMWE technology combines the advantages of AWE and PEMWE technologies, such as lower catalyst costs, high working current density, and wider load operating range, and has great application prospects in large-scale green electrolysis of water for hydrogen production. As one of the core components of the AEMWE electrolysis water device, high-performance AEM, which has high OH^−^ conductivity, good alkali stability, excellent chemical and mechanical stability, good gas barrier performance, and low preparation cost, is the key to achieving efficient hydrogen production and long-term operation of the electrolysis water device. This work provides an overview of recent studies by domestic and foreign researchers on the transport mechanism of OH^−^, as well as the degradation of cationic groups and main chains; it summarizes the modification design of different cation types and polymer skeleton structures, as well as their application progress in the field of AEMWE, providing insights for future researchers. At present, significant progress has been made in the research of AEM materials, but there are still many challenges to be overcome in order to achieve the industrial application of AEM membranes in AEMWE:

- AEMWE hydrogen production technology has garnered significant attention for combining the advantages of both AWE and PEMWE technologies. Although there have been notable advancements in the development of key components for AEM electrolyzers in recent years, such as Enapter in Germany, which has begun supplying the world’s only commercial AEM electrolyzer and hydrogen production system; its hydrogen production rate per cell is only 0.5 m^3^/h, requiring dozens of units to be connected in series to achieve a hydrogen production scale in the hundreds of kilowatts. However, due to technical barriers in key materials and manufacturing challenges, AEMWE technology remains some distance from large-scale practical application. Therefore, based on the current state of AEMWE research, the following outlook is proposed to accelerate the development and commercialization of AEMWE technology(The detailed expression is incorporated into Figure 15):
- Developing AEMs with high ionic conductivity, high strength, and high chemical stability is of paramount importance for overcoming the development bottlenecks in AEMWE hydrogen production technology. Currently, AEM research remains in its early stages. Existing domestic and international products still face challenges in balancing ionic conductivity, chemical stability, and mechanical stability under the harsh operating conditions of water electrolysis for hydrogen production. Furthermore, these products are only available in small sizes, making it difficult to meet industrial application requirements. Therefore, it is necessary to gain a deeper understanding of the degradation mechanisms of the polymer backbone and cationic groups, develop highly active cationic group/backbone/side chain structures, and construct effective ion transport pathways by regulating the bonding modes between cationic groups and the polymer backbone, thereby enhancing the ionic conductivity and stability of AEMs.
- Strengthening mechanistic and theoretical research. First, we need to conduct in-depth studies on the failure mechanisms of AEMs to uncover the root causes of their degradation and lifespan limitations. By gaining a thorough understanding of these failure mechanisms, we can specifically improve the stability and durability of membrane materials, thereby enhancing electrolysis efficiency and reliability. Second, we need to investigate the transport behavior of water molecules and gas products within the electrolyzer, studying their kinetic characteristics such as confinement and diffusion. A deeper understanding of the transport mechanisms of reactants and products will enable the design of more efficient electrolyzer systems. Third, we need to conduct in-depth research on OER/HER mechanisms to elucidate the generation mechanisms of oxygen and hydrogen during electrolysis. By investigating the active sites and reaction pathways of catalysts, it will be possible to develop more efficient and cost-effective catalysts, thereby increasing the yield of oxygen and hydrogen while reducing energy losses. However, current research on AEM failure mechanisms, water transport kinetics, and OER/HER mechanisms remains limited, and theoretical studies based on these areas are essential for breakthroughs in electrolysis technology.
- Developing high-performance electrolyzer components. To compete with existing electrolyzer technologies, in addition to the AEM, AEMWE must achieve breakthroughs in materials and technologies related to cathodes, anode electrocatalysts, ionomers, and membrane electrode assembly integration. For OER electrocatalysts, among non-transition metal elements, NiFe-based catalysts exhibit the highest activity; however, the mechanism of active sites in NiFe-based catalysts remains controversial, and further research is needed regarding their stability and potential degradation mechanisms. For HER electrocatalysts, various types of PtNi and Pt/C platinum-based catalysts still exhibit the best performance. Among non-platinum-based catalysts, NiMo- and NiCo-based catalysts show the greatest development potential, and doping with heteroatoms such as nitrogen, phosphorus, and sulfur can enhance HER activity. Strengthening research and development on bifunctional catalysts can further simplify the system, reduce costs, and enhance the competitiveness of AEMWE. The development of ionomers with high ionic conductivity and high chemical stability is also crucial; studies have found that the influence of AEI on electrochemical performance even exceeds that of the AEM. Currently, most research focuses on the development of AEMs and electrocatalysts; research on MEA characteristics needs to be improved, particularly regarding the interfaces between the catalytic layer and the membrane, as well as between the catalytic layer and the porous transport layer, whose properties remain unclear. Furthermore, improvements to the gas diffusion layer and bipolar plates are needed to enhance heat and mass transfer capabilities and battery efficiency. Efforts are also required to transition the electrolyte from weakly alkaline solutions to pure water or even seawater, thereby further reducing operational and maintenance costs.
- The issue of balancing OH^−^ conductivity with mechanical/dimensional stability. Compared to H^+^, OH^−^ has a larger size and lower diffusion rate, with a diffusion rate only about half that of H^+^. This inherent difference itself determines that preparing an AEM with high ion conductivity is more challenging compared to PEM membranes. Secondly, when AEM operates in an air environment, OH^−^ may react with CO_2_ in the air, producing carbonates or bicarbonates that can also affect ion conduction. In addition, most AEMs rely on increasing IEC to improve ion conductivity, but this inevitably sacrifices size/mechanical stability, leading to excessive membrane expansion under fully humidified conditions.
- Alkali stability issue. Due to the long-term operation of AEMWE cells under high-temperature and strong alkali conditions (60–80 °C), the presence of strong nucleophilic OH^−^ poses higher requirements for the alkali resistance and electrochemical stability of cationic groups and polymer main chains. By modifying the cation and polymer backbone, the alkali stability of AEM has been improved to a certain extent. However, the stability of AEM under actual working conditions still faces challenges.
- The issue of preparation cost. Although high-performance AEMs can be prepared through functionalization, crosslinking, and copolymerization, the complexity of raw materials and synthesis processes results in high preparation costs, limiting their applications.

Therefore, future research on AEMs can be carried out from the following aspects: designing new alkali-resistant and stable cationic groups and polymer backbones, and exploring the intrinsic mechanism of the influence of polymer backbones on the stability of cationic groups; various strategies such as crosslinking, block copolymerization, side chain grafting, and composite membrane technology to construct efficient ion transport channels and further enhance the ion conductivity of AEM should be explored; the degradation mechanism of AEM materials under actual working conditions should be explored, and theoretical guidance for designing AEM membranes that meet practical application needs should be provided.

## Figures and Tables

**Table 1:** Illustration of the main technical parameters and characteristics for alkaline water electrolysis, proton exchange membrane water electrolysis, and anion exchange membrane water electrolysis [3,4,5,32,33,34,35,36,37].

Comparison Terms	Alkaline Electrolytic Water for Hydrogen Production	Hydrogen Production through Electrolysis of Water with Proton Exchange Membrane	Hydrogen Production through Water Electrolysis with Anion Exchange Membrane
Diaphragms	Porous diaphragms (e.g., PPS woven)	Proton exchange membranes (e.g., Nafion)	Anion exchange membrane
Cathodes	NiMo alloy	Platinum group metals	Transition metal
Anodes	NiCo alloy	RuO*_x_*, IrO*_x_*	Transition metal
Polarized	Stainless steel Ni-plated	Graphite or titanium sheet	Nickel or stainless steel plate
Electrolytes	KOH solution	Purified water	Alkaline solution or purified water
Current density/(A/cm^2^)	<0.5	1~2	1~2
Operating temperature/ ℃	60~90	50~90	40~80
Gas purity	>99.5%	>99.99%	>99.99%
Lifespan/h	≈ 100,000	<10,000	<2000
Costs	Lower	High	-
Technology maturity	Maturity	Small-scale commercialization	Under development
Vantage	High technological maturity; low cost using non-precious metal catalysts	High energy efficiency; high gas purity; high current density; rapid response	High energy efficiency; fast response; low cost non-precious metal catalysts; high gas purity
Drawbacks	Low energy efficiency; low current density; low gas purity; poor responsiveness	Precious metal catalysts and Nafion membranes, high cost; poor stability	Low technological maturity; short lifespan

**Table 2:** AEMWE technology status and future key performance indicators [37,38,39,40,41].

Indicators	2020	2050	R&D Priorities
Normal current density/(A/cm^2^)	0.2–2	>2	Membranes, electrocatalyst
Voltage range/V	1.4–2.0	<2	Electrocatalyst
Scope of work/°C	40–60	80	Membranes, electrocatalyst
Battery pressure/MPa	<3.5	>7.0	Membranes
Load range/%	5–100	5–200	Membranes
H_2_ purity/%	99.9–99.999	>99.9999	Membranes
Electrolytic efficiency/%	52–67	>75	Electrocatalyst
Power consumption for hydrogen production (fuel cell stack)/(KW·h/kg)	51.5–66	<42	Electrocatalysts, membranes
Power consumption for hydrogen production (system)/(KW·h/kg)	57–69	<45	Hydrogen plant balancing
Life cycle (fuel cell stack)/h	>5000	100,000	Membrane, electrode
Stack cost (≥1 MW)/(USD/KW)	Unknown	<100	Membrane electrode
System cost (≤10 MW)/(USD/KW)	Unknown	<200	Rectifier

## Data Availability

No new data were created or analyzed in this study.
