# Correction: Emin et al. Recent Progress in Anion Exchange Membrane Water Electrolysis: From Membrane Materials to System Components. *Membranes* 2026, *16*, 185

**DOI:** 10.3390/membranes16110377

**Published:** 2026-11-05

**Authors:** Adil Emin, Jiarui Liu, Xian Sun

**Affiliations:** School of Chemical Engineering, Xinjiang University, Urumqi 830017, China; 18191859848@163.com

In the publication [1], following consultation and unanimous agreement among all authors, Hao Jiang has requested to be removed as an author. His decision was motivated by the perception that his contribution to the work was insufficient. The revised authorship and affiliations are as follows:

Adil Emin *, Jiarui Liu and Xian Sun *

School of Chemical Engineering, Xinjiang University, Urumqi 830017, China; 18191859848@163.com

**Author Contributions:** A.E.: writing—original draft preparation, research work curation, formal analysis, investigation, and supervision. J.L.: research work curation, formal analysis, and investigation. X.S.: formal analysis, investigation, and supervision. All authors have read and agreed to the published version of the manuscript.

The authors state that the scientific conclusions are unaffected. This correction was approved by the Academic Editor. The original publication has also been updated.
